# A knockdown gene approach identifies an insect vector membrane protein with leucin-rich repeats as one of the receptors for the VmpA adhesin of flavescence dorée phytoplasma

**DOI:** 10.3389/fcimb.2023.1289100

**Published:** 2023-11-06

**Authors:** Francesca Canuto, Sybille Duret, Marie-Pierre Dubrana, Stéphane Claverol, Sylvie Malembic-Maher, Xavier Foissac, Nathalie Arricau-Bouvery

**Affiliations:** ^1^ Univ. Bordeaux, INRAE, Biologie du Fruit et Pathologie, UMR 1332, Villenave d’Ornon, France; ^2^ Univ. Bordeaux, Bordeaux Proteome, Bordeaux, France

**Keywords:** *Euscelidius variegatus*, phytoplasma, protein-protein interaction, LRR proteins, VmpA, RNAi

## Abstract

**Introduction:**

The adhesion of flavescence dorée phytoplasma to the midgut epithelium cells of their insect vectors is partially mediated by the variable membrane protein A (VmpA), an adhesin which shows lectin properties. In order to identify the insect receptor for VmpA, we identified *Euscelidius variegatus* cell proteins interacting with recombinant VmpA-His_6_.

**Methods:**

The *E. variegatus* proteins were identified by mass spectrometry analysis of VmpA-*E. variegatus* protein complexes formed upon *in vitro* interaction assays. To assess their impact in VmpA binding, we reduced the expression of the candidate genes on *E. variegatus* cells in culture by dsRNA-mediated RNAi. The effect of candidate gene knockdown on VmpA binding was measured by the capacity of *E. variegatus* cells to bind VmpA-coated fluorescent beads.

**Results and discussion:**

There were 13 candidate proteins possessing potential N-glycosylation sites and predicted transmembrane domains selected. The decrease of expression of an unknown transmembrane protein with leucine-rich repeat domains (uk1_LRR) was correlated with the decreased adhesion of VmpA beads to *E. variegatus* cells. The uk1_LRR was more expressed in digestive tubes than salivary glands of *E. variegatus*. The protein uk1_LRR could be implicated in the binding with VmpA in the early stages of insect infection following phytoplasmas ingestion

## 1 Introduction

Phytoplasmas are cell wall-less bacteria characterized by their ability to colonize two hosts belonging to two different kingdoms, their plants host and their insect vector (Lee et al., 2000; Gross et al., 2022). Phytoplasmas are responsible for severe diseases in a large number of cultivated and ornamental plants and are transmitted by leafhoppers, planthoppers, and psyllids (Weintraub and Beanland, 2006). The control of phytoplasma-associated diseases relies on prophylactic measures based on disease surveillance, elimination of infected plants in both production fields and nurseries, and insecticide treatments against the vector insects when recommended (Weintraub and Wilson, 2009; Jeger et al., 2016; Oliveira et al., 2019; European Food Safety Authority (EFSA) et al., 2020). Phytoplasmas perform a persistent propagative cycle into their insect vectors where they must invade and cross two main barriers, the midgut epithelium and salivary gland cells (Maillet and Gouranton, 1971). Thus, after their ingestion with infected sap, adhesion of phytoplasmas to the apical surface of vector intestinal cells constitutes an initial and critical step in insect invasion. Once this first barrier is crossed, the adhesion of phytoplasmas to salivary gland cells is as well critical for transmission to the plant achieved upon injection of infected saliva. A given phytoplasma has one or few insect vectors depending on the level of specificity of the interaction. Insights into the ecology and populations genetic of the flavescence dorée phytoplasmas (FDp) have revealed the correlation between vector specificity of phytoplasma strains and positively selected variant sequences of phytoplasma variable membrane proteins VmpA and VmpB (Malembic-Maher et al., 2020). It is now known that VmpA is acting as an adhesin that specifically interacts with glycoconjugates at the surface of insect vector cells (Arricau-Bouvery et al., 2018; Arricau-Bouvery et al., 2021). VmpA receptors on the surface of host cells are essential for phytoplasma colonization of insect cells, and variation in insect glycoproteins might be responsible for insect vector specificity. Once attached to insect cells, FD phytoplasmas enter cells by clathrin-mediated endocytosis, a process important for colonization of its insect vector (Arricau-Bouvery et al., 2023). Identifying VmpA receptors could provide new insight in the mechanism of entry of the FD phytoplasma into insect cells. Moreover, in a context of pesticide reduction, this knowledge could help in developing new strategies of disease control by blocking the phytoplasma–insect cell interaction as it could be achieved for the phloem-sap pest “*Candidatus* Liberibacter asiaticus” (Merfa et al., 2022; Guo et al., 2023).

Reducing the expression of VmpA receptors in the leafhopper cells in culture should be correlated with a decrease in their ability to bind VmpA. Knockdown gene expression is a powerful tool to elucidate gene function and can be achieved by long double-stranded RNA (dsRNA) introduced into the organism or the cells (Baum et al., 2007; Mondal et al., 2020). In RNA interference (RNAi), the long dsRNAs are processed in small interfering RNAs (siRNAs) that trigger degradation of the complementary target mRNA. RNAi in mammal cells is performed using siRNA, whereas in insects it is generally performed using long double-stranded RNA (Jackson et al., 2003). This approach had been used for instance to inactivate genes in several *Drosophila* cells in culture (Clemens et al., 2000; Echeverri and Perrimon, 2006). RNAi was also used with success in leafhoppers and whiteflies *via* injection or ingestion of dsRNAs (Abbà et al., 2019; Jain et al., 2022).

The objective of this study was to design a strategy to identify VmpA receptors. We first identified by mass spectrometry proteins present at the surface of *Euscelidius variegatus* cell line Euva-11 that potentially interact with the adhesin VmpA by purification on affinity column and far-western blot assays. Once these receptor candidates were identified, we intended to screen the candidates by measuring the effect of a decrease of their expression by *E. variegatus* cells in culture on their ability to bind VmpA-coated latex beads. We optimized RNAi to knockdown simultaneously up to 13 genes coding these candidates in the *E. variegatus* cell line Euva-11.

## 2 Materials and methods

### 2.1 Insect rearing, cell lines, and phytoplasma isolate

Healthy *E. variegatus* leafhoppers were originally collected in Villenave d’Ornon, France. They were reared in cages on broad beans *Vicia faba* var. aquadulce and oats *Avena sativa* from seeds purchased from Castros Gerand and Jardiland, respectively, at 25°C in a greenhouse under a 16-h light/8-h dark photoperiod. To obtain infected *E*. *variegatus*, L4–L5 nymphs were transferred by groups of 100 on FDp-infected broad bean for phytoplasma acquisition. Seven days later, *E*. *variegatus* were placed in cages on healthy broad bean for a latency period of 3–4 weeks. This study complies with relevant institutional, national, and international guidelines and legislation.

The *E*. *variegatus* Euva-11 cell line was established from embryos of *E*. *variegatus* (Arricau-Bouvery et al., 2018). In brief, the eggs were sterilized with bleach solution and then with 70% ethanol. After rinsing, the eggs were ground in culture medium made of 350 mL Schneider’s Drosophila medium (Invitrogen), 100 mL Grace’s insect cell culture medium (Invitrogen), 50 mL heat-inactivated fetal bovine serum (Eurobio), and 2 mL G-5 supplement (Invitrogen). The Euva-11 cells were cultivated at 25°C. After the first colonies developed and the cell line was established, the cells were passed using trypsinization every week with an additional change in modified culture medium made of 350 mL Schneider’s Drosophila medium, 100 mL Grace’s insect cell culture medium, and 50 mL heat-inactivated fetal bovine serum during the week.

The phytoplasma strain FD92 was originally transmitted to broad bean by infected *Scaphoideus titanus* sampled in FD-diseased vineyards in southwest France (Caudwell et al., 1970; Angelini et al., 2001). FDp was then continuously maintained in broad beans by *E*. *variegatus* transmission according to a published protocol (Caudwell et al., 1972).

### 2.2 Identification of candidate insect proteins

#### 2.2.1 Purification of VmpA–*E. variegatus* protein complexes on affinity column

Three 75-cm^2^ flasks of *E*. *variegatus* Euva cells were trypsinized. The cellular pellet obtained from 54 mL of culture was washed once with PBS 1× and then resuspended in 100 µL in Rx buffer [0.1% Triton X-100, 100 mM KCl, 3 mM NaCl, 3.5 mM MgCl_2_, 1.25 mM EGTA, 10 mM Hepes, pH 7.3 (Suzuki et al., 2006; Galetto et al., 2011)]. The sample was vortexed, incubated at room temperature for 5 min, and centrifuged for 3 min at 14,000 g. The supernatant of Euva proteins was then recovered, and the concentration of proteins was measured using the Bradford assay (Bio-Rad). Resuspended proteins (1.3 mg) were incubated overnight at 4°C with 1 mg of the recombinant protein VmpA-His_6_ of the FD92 phytoplasma (see Section 2.5) and protease inhibitor cocktail (Sigma-Aldrich). Then, the mixture was purified on an affinity nickel column using the ÄKTA™ system (GE Healthcare, US). As a negative control, a purification on the affinity nickel column was performed using 1.3 mg of Euva cell supernatant proteins in the absence of VmpA.

#### 2.2.2 Protein separation by SDS-PAGE electrophoresis and far-western blot analysis

The pellet of trypsinized Euva cells resulting from the extraction with Rx buffer described previously was resuspended in Rx-T-DOC buffer (1% Triton X-100, 0.5% DOC, 100 mM KCl, 3 mM NaCl, 3.5 mM MgCl_2_, 1.25 mM EGTA, 10 mM Hepes, pH 7.3), vortexed, and then incubated at 4°C on an orbital shaker for 1 h to solubilize membrane proteins. A centrifugation of 3 min was performed at a speed of 14,000 g at a temperature of 4°C. The supernatant containing an extract enriched in membrane proteins was then recovered and the pellet directly mixed with Laemmli buffer. In summary, the proteins were separated into three fractions, the proteins soluble in Rx fraction, the Rx-T-DOC soluble fraction, and the insoluble fraction, *i.e.*, pellet suspended directly in Laemmli buffer. An amount of 30 µg of proteins present in the Rx supernatant fraction and the equivalent in volume of supernatant recovered with Rx-T-DOC were suspended in Laemmli buffer to a final volume of 26 µL. Samples were boiled just before being separated by SDS-PAGE electrophoresis on two equivalent 10% acrylamide gels. Proteins from the first gel were then transferred on a nitrocellulose membrane for 1.25 h at 70 V and then stained with red Ponceau solution to assess the quality of protein transfer. The membrane was washed with purified water and then saturated with a blocking buffer consisting of a solution of PBS containing 5% of skimmed milk for 4 h at room temperature. After saturation, the membrane was incubated overnight at 4°C on an orbital shaker with the protease inhibitor cocktail (1/1,000) (Sigma-Aldrich) and the recombinant protein VmpA-His_6_ at a concentration of 45 µg mL^−1^. The membrane was then washed in PBS then in PBS + 0.2% Tween 20 prior to incubation with a primary anti-VmpA monospecific polyclonal antibody diluted 1:7,000 (Covalab). After incubation, the membrane was washed with PBS then with PBS + 0.2% Tween 20 and incubated with the secondary antibody anti-IgG rabbit-HRP (Sigma-Aldrich). After washing with PBS, the membrane was incubated with HRP substrate and visualized through the ChemiDoc Imaging System (Bio-Rad).

The second gel was stained with Coomassie blue. The bands corresponding to the signals observed on the far-western blot anti-VmpA were excised and digested with trypsin as previously described (Killiny 2006 Identification of a *Spiroplasma citri* hydrophilic protein associated with insect transmissibility. Microbiol. Read. Engl. 152, 1221–1230). The resulting digestion was analyzed by liquid chromatography tandem mass spectrometry (LC-MS/MS) as routinely performed on the proteomics platform of the University of Bordeaux, France, and sent to a Bordeaux proteomic platform to perform a mass spectrometry analysis.

#### 2.2.3 Mass spectrometry analysis (nLC-MS/MS)

Search parameters were as follows: mass accuracy of the monoisotopic peptide precursor and peptide fragments was set to 10 ppm and 0.02 Da, respectively. Only b- and y-ions were considered for mass calculation. Oxidation of methionines (+16 Da), methionine loss (−131 Da), methionine loss with acetylation (−89 Da), and protein N-terminal acetylation (+42 Da) were considered as variable modifications, whereas carbamidomethylation of cysteines (+57 Da) was considered as fixed modification. Two missed trypsin cleavages were allowed. Peptide validation was performed using the Percolator algorithm (Käll et al., 2007), and only “high confidence” peptides were retained corresponding to a 1% false positive rate at the peptide level. Spectra from peptides higher than 5,000 Da or lower than 350 Da were rejected. The precursor detector node was included.

#### 2.2.4 Database search and results processing

Data were searched by SEQUEST through Proteome Discoverer 2.5 (Thermo Fisher Scientific Inc.) against a SwissProt protein database (version 2022-01; 478,954 entries) and a collection of peptide sequences originating from the conceptual translation of RNA-seq data from *E. variegatus*. The dataset analyzed during the current study are available in the Transcriptome Shotgun Assembly database on NCBI: *E. variegatus*, transcriptome shotgun assembly repository, GenBank accession: GFTU00000000.1.

For the proteins selected after chromatography affinity and identified through mass spectrometry analysis, a first selection step consisted in the determination of VmpA specific retention ratio using the following equation:


(« VmpA column » abundance – « control column » abundance) ∗ 100/« VmpA column » abundance

The proteins were classified based on this value. A threshold of 90% of VmpA-specific retention was set in order to eliminate the proteins showing an affinity for the column itself. A gene ontology search was then performed in order to categorize the proteins based on their biological role.

Proteins identified by mass spectrometry analysis performed on excised bands were first selected restraining the results to peptides specifically matching sequences of the *E. variegatus in silico* predicted proteome. A second selection based on the molecular weight of the proteins followed, according to the signal observed in the far-western blot comparing the positions of bands to the ladder. As the signal observed on the far-western blot was common to the soluble and insoluble fractions, the analysis of bands G and H was restricted to proteins identified in both bands.

The parameters considered to further select the VmpA putative targets were the presence of potential N-glycosylation sites predicted by the prediction tool Glycomine (glycomine.erc.monash.edu.) and the prediction of NxS/T pattern, and the presence of predicted transmembrane domains using TMHMM v2.0 software (Krogh et al., 2001) in the amino acidic sequence.

### 2.3 Knockdown gene approach to validate interaction between VmpA and insect proteins

#### 2.3.1 Cloning of *E. variegatus* CDS

RNAs were extracted using TRIzol reagent (Thermo Fisher Scientific) under manufacturer instructions including a DNAse treatment for 1 h at 37°C. Reverse transcription was performed on 1 µg of *E. variegatus* RNA as template using SuperScript IV (Thermo Fisher) and random hexamer primers (Thermo Fisher) according to the manufacturer’s instruction. The inserts were amplified by PCR and cloned into pGEM^®^-T Easy vector (Promega) or pMINI-T2 (NEB) using primers listed in **Table S1**. Plasmids were propagated in competent *E. coli* DH10β cells. The presence of the insert was checked through sequencing by Sanger technology (GENEWIZ, Azenta) using the primers matching flanking region on the plasmid. The sequence analysis and alignment onto reference sequences (TSA *Euscelidius variegatus* taxid:13064*)* were performed using ApE v3.1.3 (Davis and Jorgensen, 2022).

#### 2.3.2 RNA interference on Euva-11 cells, RNA extraction, and mRNA analysis

The putative VmpA targets and GFP sequences were amplified by PCR from *E. variegatus* cDNA as a template using sequence-specific primers 5′ extended with 20 bp of T7 RNA polymerase promoter (**Table S1**). The HiScribe™ T7 High Yield RNA Synthesis Kit (New England Biolabs) was used on the PCR products to obtain the dsRNAs. dsRNAs were ethanol precipitated in the presence of sodium acetate 0.3 M and were resuspended in 20 µL of RNAse-free water. The purity and integrity of dsRNAs were determined through migration on agarose gel electrophoresis of 1/10 and 1/100 dilutions of the transcription products.

Euva cells were cultivated in 24-well plates (Falcon) to nearly 80% confluence and transfected with 1 µg dsRNA using FuGENE HD Transfection Reagent (Promega). At the end of incubation time of 3 or 7 days, the transfected Euva cells were collected using TRIzol Reagent (Thermo Fisher Scientific) and then stored at −20°C until extraction which was performed according to the manufacturer’s instructions. cDNAs of the samples were obtained using SuperScript™ III Reverse Transcriptase (Thermo Fisher Scientific) and 2.5 µM random hexamer primers (Invitrogen). Real-time PCR was performed on the LightCycler 480 Real-Time PCR system (Roche Diagnostics Corp) using sequence-specific primers (**Table S1**) and N′,N′-dimethyl-N-[4-[(E)-(3-methyl-1,3-benzothiazol-2-ylidene)methyl]-1-phenylquinolin-1-ium-2-yl]-N-propylpropane-1,3-diamine (SYBR Green, Roche). The cycling programs for all amplifications was 95°C for 15 min, followed by 40 cycles of 95°C for 30 s, 60°C for 30 s, and 72°C for 30 s. The two housekeeping genes glutathione S-transferase (GST) and tubulin β (tubβ) served as reference genes for mRNA quantification. The relative fold change in gene expression was calculated using the 2^−ΔΔCt^ method (Livak and Schmittgen, 2001).

#### 2.3.3 Adhesion assays of VmpA-His_6_-coated beads to Euva cells

The recombinant VmpA-His_6_ proteins were obtained as previously described (Arricau-Bouvery et al., 2018). In brief, the proteins were expressed in *E*. *coli* BL21 Star (DE3) cells containing the plasmid pET28-VmpA-His_6_ and purified on a His-select nickel affinity gel packed column (Sigma) according to the manufacturer’s protocol. Red fluorescent latex beads exposing ammine at their surface (4 × 10^9^ beads at 1 µm, Sigma-Aldrich) have been coated with the recombinant VmpA-His_6_. The beads were washed with MES buffer 50 mM pH 6.1 and then incubated with recombinant proteins at a final concentration of 9 µM of VmpA-His_6 _+ 1 µM BSA. EDAC 16 mM was added to activate the reaction. The beads were then incubated for 2 h in the dark on an orbital shaker. After a 3-min centrifugation, the coated beads have been washed once with MES buffer 50 mM pH 6.1 and then resuspended in 200 µL of the same buffer. The coated beads were stored in the dark at 4°C up to 7 days. Coating of the beads was verified for each experiment performing a dot blot using a rabbit antibody anti-VmpA.

Three days post transfection with dsRNA, to allow the turnover of the target proteins (Clemens et al., 2000), Euva cells on coverslip in 24-well plates were washed twice with Schneider’s medium (Gibco). VmpA-His_6_-coated beads were diluted in Schneider’s medium (1/100), and 100 µL was dispensed on the cells. The cells were then incubated at 25°C for 1 h.

#### 2.3.4 Immunofluorescent staining and microscopic observations

After incubation, cells were washed twice with Schneider medium and then twice with PBS 1× (Eurobio Scientific). The cells were then fixed using paraformaldehyde at a final concentration of 4% (Electron Microscopy Sciences) during 15 min. The fixed cells were washed for three times with PBS 1× then in distilled water and stained with DAPI 1 mg mL^−1^ during 5 min. The cells were then washed in water and mounted in ProLong Gold Antifade reagent (Invitrogen). Then, the cells were imaged using a Zeiss Axio Imager epifluorescence microscope with an objective with a numerical aperture of 1.4. The filters used for excitations and emissions were BP 377/50 and BP 447/60 for DAPI and BP 562/40 and LP 593 for red fluorescent beads. For Z-stack acquisition, images were acquired every 0.3 µm from the bottom to the top to Euva cells and Z-projections were performed before counting of adherent beads with the free software ImageJ (v. 1.53f) (Schindelin et al., 2012). There were 20 random positions captured for each treatment.

The Euva cells cultivated in a 24-well plate were observed with the inverted microscope Eclipse TS100 (Nikon) at 3 days post transfection, prior to RNA extraction. Images were taken in random positions of the well at magnifications of 4, 10, and 20.

### 2.4 Assessment of uk1_LRR expression on *E. variegatus*

Non-infected *E*. *variegatus* young adults sampled from a synchronized rearing were dissected at the stereomicroscope. Male and female tissues were analyzed separately. Seven salivary glands from females and 7 from males, 7 midgut organs from females and 11 from males, and 6 ovaries with eggs were recovered, pooled to have a sufficient amount of biological material to analyze and kept in TRIzol. Total RNA extraction was performed as previously described, and relative quantification of *uk1_LRR* transcripts was assessed through real-time RT-PCR. Glutathione S-transferase (GST), tubulin β (tub β), and elongation factor 1 (EF1) were used as reference genes to normalize expression values.

A total of 240 nymphs of stages 4 and 5 were put on broad beans to obtain a synchronized rearing. After 10 days, half of young insects were transferred onto healthy broad beans while the other half were transferred onto broad beans infected with FDp for acquisition. After 7 days, eight insects were sampled for each condition and the remaining insects were transferred onto healthy broad beans for latency. Eight insects were sampled every 7 days up to 40 days after beginning of acquisition. The head and abdomen of each insect were analyzed separately. Total RNA was extracted as described previously. Relative quantification of *uk1_LRR* transcripts was assessed through real time RT-PCR as well as the expression of FDp tuf gene hereby used as a proxy of FDp titer and transcriptional activity*. E. variegatus* glutathione S-transferase (GST) was used as reference gene to normalize expression values.

## 3 Results

### 3.1 Selection of transmembrane Euva proteins interacting with VmpA retained in the affinity column

**Figure 1:**
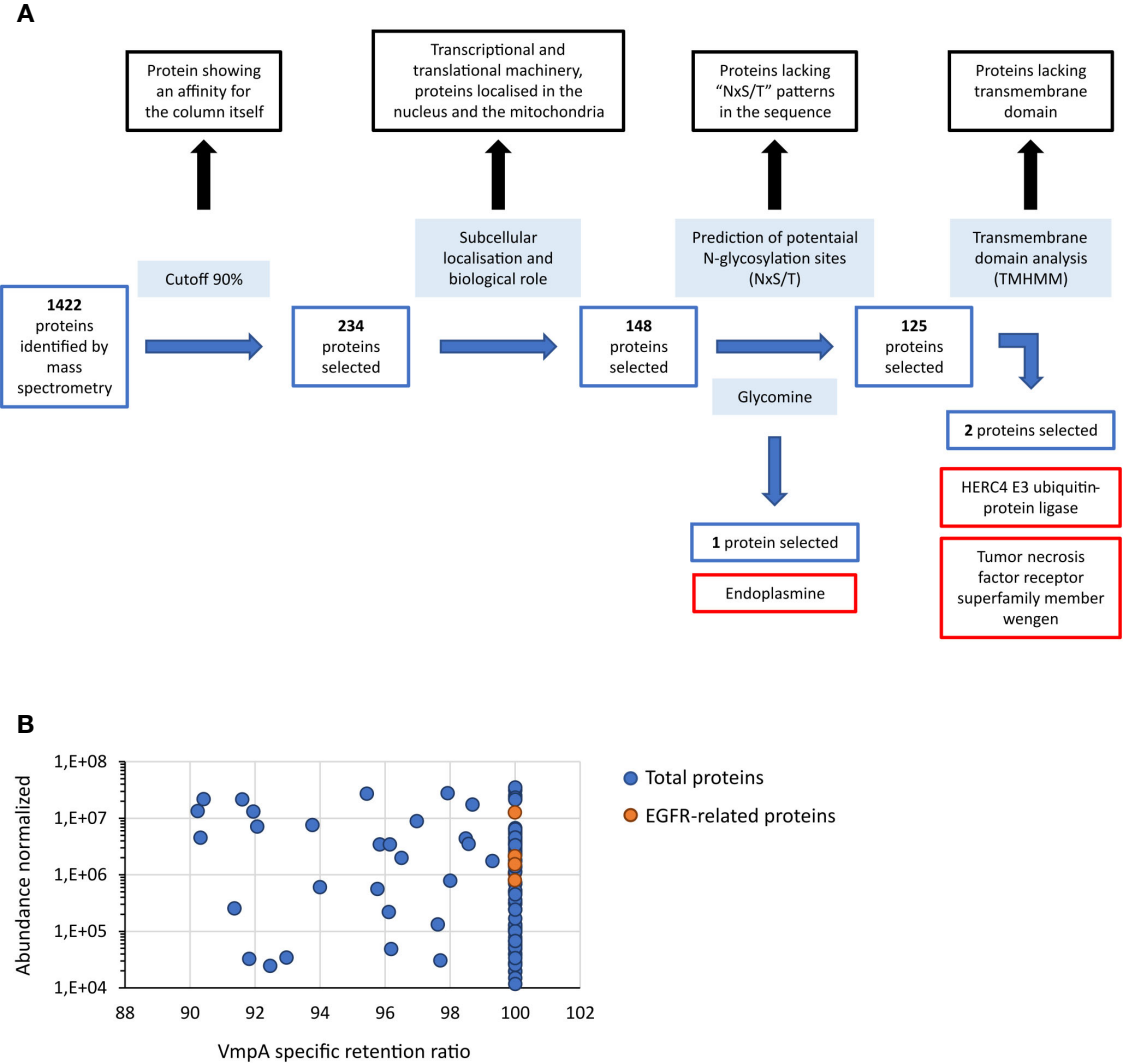
Proteins of Euva cells that interact with VmpA-His_6_. **(A)** Process of selection of candidates that interact with VmpA-His_6_. The different steps of the process are indicated inside light-blue boxes. The proteins without interest are indicated in boxes lined with black. The proteins selected at each step are indicated in boxes lined with blue, and the name of the three proteins finally selected are in boxes lined with red. **(B)** Abundance of the proteins interacting with VmpA-His_6_ selected with a retention ratio superior to 90%. The five proteins interacting with EGFR and implicated in its recycling process are indicated in orange.

As VmpA interacted with proteins of the cell lines Euva established from *E. variegatus*, Euva proteins were purified using an affinity column consisting of VmpA-His_6_ bound to nickel-resin and eluted proteins were identified by mass spectrometry (nLC-MS/MS). The control consisted of purification of the Euva proteins on the Ni-NTA column. Mass spectrometry analysis identified 1,422 proteins among proteins binding VmpA-His_6_ retained by the column and 1,604 for the control condition (Euva proteins only). A cutoff of 90% was applied to the specific VmpA retention ratio which allowed the selection of 239 proteins (**Figure 1A**). They were classified based on the KEGG classification (**Table 1**). Proteins belonging to the transcriptional and translational machinery and proteins localized in the nucleus and mitochondria were removed from the pool of protein candidates that could interact with VmpA. The 148 remaining proteins were analyzed based on the presence of predicted glycosylation sites using Glycomine software that selected only the endoplasmin (endo) protein (one site with *p* = 0.958 and four sites with *p* > 0.8). We additionally searched the “NxS/T” glycosylation site patterns in the amino acidic sequence of the identified proteins and selected 125 candidates. Among them, the transmembrane segments were identified using TMHMM 2.0 software and only two membrane proteins were selected, the HERC4 E3 ubiquitin-protein ligase (HERC4) and the tumor necrosis factor receptor super family member wengen (wengen). Interestingly, five proteins that are known to be implicated in the turnover of the epidermal growth factor receptor (EGFR) were found among the pool of 148 proteins (**Figure 1B**). Despite the absence of the EGFR in this pool and because it is a 92kDa glycosylated protein present at the surface of almost all cells, we decided to additionally test EGFR as a fourth candidate. Indeed, it has been shown previously that VmpA-His_6_ interacted essentially with Euva proteins with apparent masses of 90–95 kDa (Arricau-Bouvery et al., 2021). To test the effect of gene expression knockdown on the interaction between these four candidates and the protein VmpA, the production of these candidates within Euva cells was inhibited by RNA interference (RNAi) and VmpA adhesion assays were performed and are detailed below.

**Table 1:** Repartition of the 239 Euva proteins that interacted with VmpA-His_6_ in relation to their KEEG classification.

KEEG classification	Number of proteins
Global cellular processes	142
Cytoskeleton	18
Vesicular trafficking	17
Binding to membrane/to membrane receptor	9
Other	53

### 3.2 Selection of transmembrane Euva proteins interacting with VmpA using far-western assay

**Figure 2:**
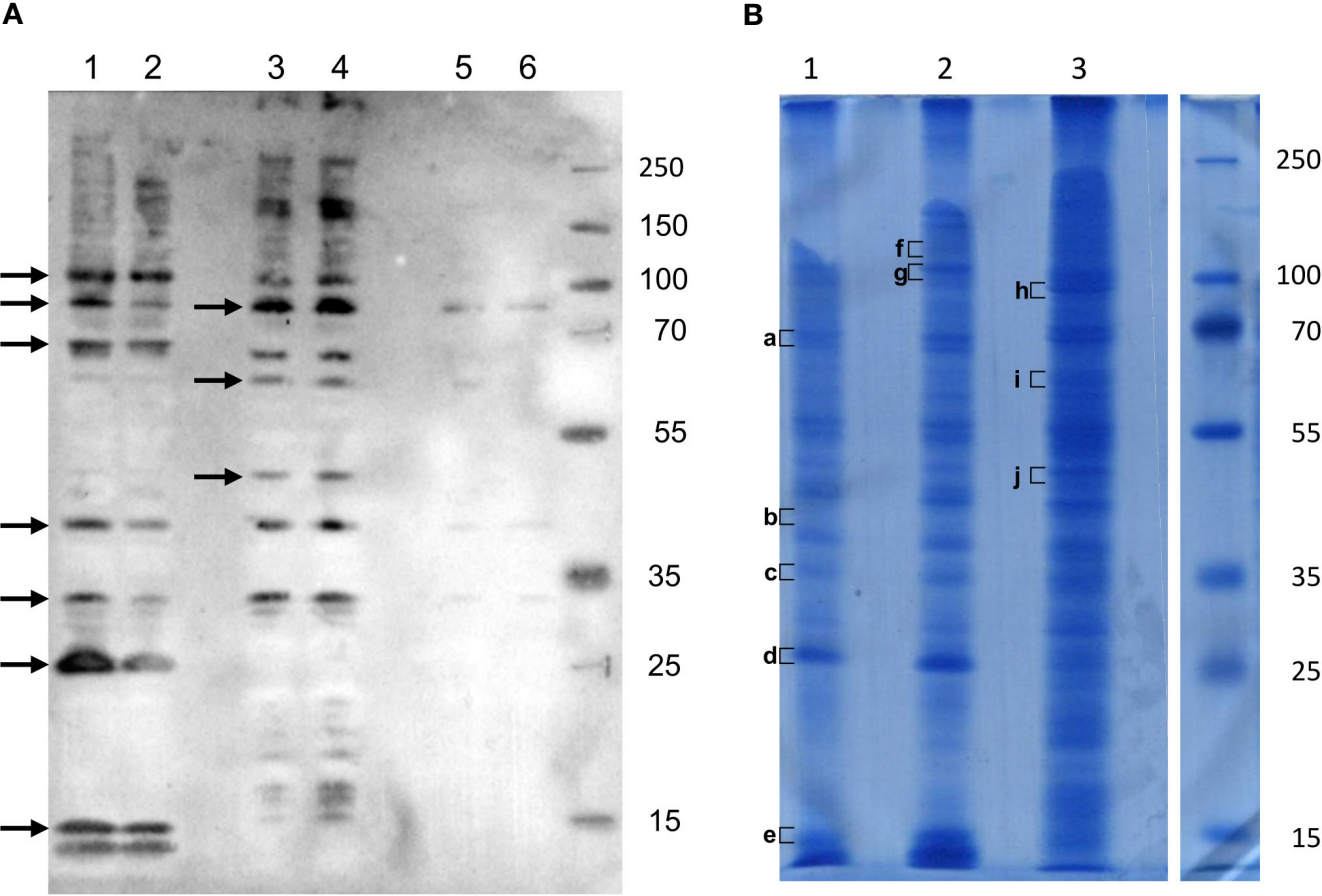
Interaction of VmpA-His_6_ with Euva cell proteins *in vitro*. **(A)** Euva proteins separated on SDS PAGE and transferred to the nitrocellulose membrane were incubated with recombinant VmpA-His_6_. Arrows indicated the insect proteins selected to be extracted from SDS PAGE gel indicated with square bracket in B **(B)** Euva proteins colored with Coomassie blue. Euva-12 (1) and Euva-11 (2) proteins present in the pellet (insoluble fraction). Euva-12 (3) and Euva-11 (4) proteins soluble in Rx-T-DOC buffer. Euva-12 (5) and Euva-11 (6) proteins soluble in the Rx buffer. a to j, bands of proteins that were analyzed with nLC-MS/MS.

To better enrich protein fractions in transmembrane proteins, we increased the concentration of Triton X-100 and added sodium deoxycholate to the Rx buffer (Rx-T-DOC). The two cell lines Euva-11 and Euva-12 that showed different cell morphologies and therefore could express different proteins on their surface interacting with VmpA were used for the protein extraction process. The Euva-11 cell line was more homogenous and mostly contained cells that looked like epithelial cells. The Euva-12 cell line was more heterogenous but also contained epithelial-like cells. The different fractions of proteins extracted from Euva cells were submitted to far-western blot using VmpA-His_6_ for interaction (**Figure 2A**). Even if the two cell lines did not fully look the same, no difference was observed between the VmpA-His_6_ interaction patterns of Euva-11 proteins and Euva-12 proteins. The profile of proteins that interacted with VmpA-His_6_ was quite similar between the different extracted fractions but for some bands the intensity of the signal observed was increased (**Figure 2A**, arrows). Seven main bands were visible in the fraction submitted to Rx-T-DOC extraction and eight in the insoluble fraction. Four bands were in common between insoluble and soluble fractions, in detail bands of 32, 45, 90, and 110 kDa. In parallel of the far-western blot, Euva proteins with masses corresponding to those observed on far-western blot were excised from a Coomassie blue-stained gel and underwent nLC-MS/MS mass-spectrometry analysis. The bands of proteins that were removed from the SDS PAGE gel are indicated by square brackets, as shown in **Figure 2B**. A total of 205 proteins were selected because they both have TMHMM-predicted transmembrane segments and “NxS/T” pattern(s) (**Table 2**, details in **Table S2**). Among these proteins we selected nine proteins that were predicted to be mostly localized at the surface of the cell according to TMHMM prediction (**Tables 2**, **3**).

**Table 2:** Repartition of Euva proteins extracted with the buffer Rx Triton X-100 1% + DOC 0.5% and that interacted with VmpA-His_6_.

Band	Property	Number of proteins	Number of proteins selected by				Protein selected for adhesion assays	Candidates abbreviation
			Weight (range of selection)		Transmembrane segment (TMHMM)	NxS/T pattern		
a	Insoluble	1,295	340	(60–80 kDa)	21 (6%)	21	Lysosome membrane protein 2 [Homalodisca vitripennis] Protein cueball isoform X1 [Homalodisca vitripennis]	CD36-like Cueball
b	Insoluble	1,446	333	(40–50 kDa)	23 (7%)	17		
c	Insoluble	950	280	(30–40 kDa)	17 (6%)	14		
d	Insoluble	886	240	(20–30 kDa)	17 (7%)	16		
e	Insoluble	322	19	(13–15 kDa)	1 (5%)	1		
f	Insoluble	1,077	153	(100–120 kDa)	13 (8%)	13	Transforming growth factor-beta-induced protein ig-h3-like [Homalodisca vitripennis] Protein draper isoform X1 [Zootermopsis nevadensis]	Fasciclin Draper
g+h	Insoluble + soluble	684	133	(85–110 kDa)	14 (11%)	14	Protein containing LRR domains (identification *via* InterPro) integrin β Sodium/calcium exchanger 3-like isoform X1 [Homalodisca vitripennis] Transforming growth factor-beta-induced protein ig-h3-like [Homalodisca vitripennis] Leucine-rich repeat-containing protein 15-like [Homalodisca vitripennis]	uk1_LRR Integrin β Na/Ca exchanger Fasciclin uk2_TLR
i	Soluble	1,441	421	(55–68 kDa)	58 (14%)	54	Protein cueball isoform X1 [Homalodisca vitripennis] Lysosome membrane protein 2 [Homalodisca vitripennis]	Cueball CD36-like
j	Soluble	1,222	462	(40–55 kDa)	63 (14%)	55	Uncharacterized protein LOC124356975 [Homalodisca vitripennis]	uk3

The proteins were selected sequentially by their weight, the presence of predicted transmembrane domain using TMHMM, and the presence of “NxS/T”-indicated potential site of glycosylation. The proteins selected for adhesion assays were indicated with their abbreviation.

**Table 3:** Characteristics of the Euva proteins selected for adhesion assays.

Band	TSA *Euscelidius variegatus *(NCBI)	Sequence (BLASTP)	Abbreviation	Predicted transmembrane segments (TMHMM)	NxS/T	MW (kDa) protein
a	ORF01214079_GFTU01004399.1_00136_01779_f1	Lysosome membrane protein 2 [Homalodisca vitripennis]	CD36-like	2	6	61
	ORF03010044_GFTU01011408.1_00185_01996_f2	Protein cueball isoform X1 [Homalodisca vitripennis]	Cueball	1	6	67
f	ORF02093016_GFTU01007852.1_00001_02736_f1	Transforming growth factor-beta-induced protein ig-h3-like [Homalodisca vitripennis]	Fasciclin	1	5	94
	ORF03898939_GFTU01016123.1_00223_03282_f1	Protein draper isoform X1 [Zootermopsis nevadensis]	Draper	1	11	109
g+h	ORF03757228_GFTU01015247.1_00222_02681_f3	Protein containing LRR domains (identification *via* InterPro)	uk1_LRR	1	14	87
	ORF03985247_GFTU01016611.1_00180_02645_f3	integrin beta	integrin β	1	7	91
	ORF03874906_GFTU01015965.1_00117_02747_f3	Sodium/calcium exchanger 3-like isoform X1 [Homalodisca vitripennis]	Na/Ca exchanger	12	6	95
	ORF02093016_GFTU01007852.1_00001_02736_f1	Transforming growth factor-beta-induced protein ig-h3-like [Homalodisca vitripennis]	Fasciclin	1	5	94
	ORF03639633_GFTU01014488.1_00117_02468_f3	Leucine-rich repeat-containing protein 15-like [Homalodisca vitripennis]	uk2_TLR	1	7	82
i	ORF03010044_GFTU01011408.1_00185_01996_f2	Protein cueball isoform X1 [Homalodisca vitripennis]	Cueball	1	6	67
	ORF01214079_GFTU01004399.1_00136_01779_f1	Lysosome membrane protein 2 [Homalodisca vitripennis]	CD36-like	2	6	61
j	ORF00560024_GFTU01001746.1_00401_01750_f2	Uncharacterized protein LOC124356975 [Homalodisca vitripennis]	uk3	1	2	48

### 3.3 RNAi knockdown gene expression on Euva cells

**Figure 3:**
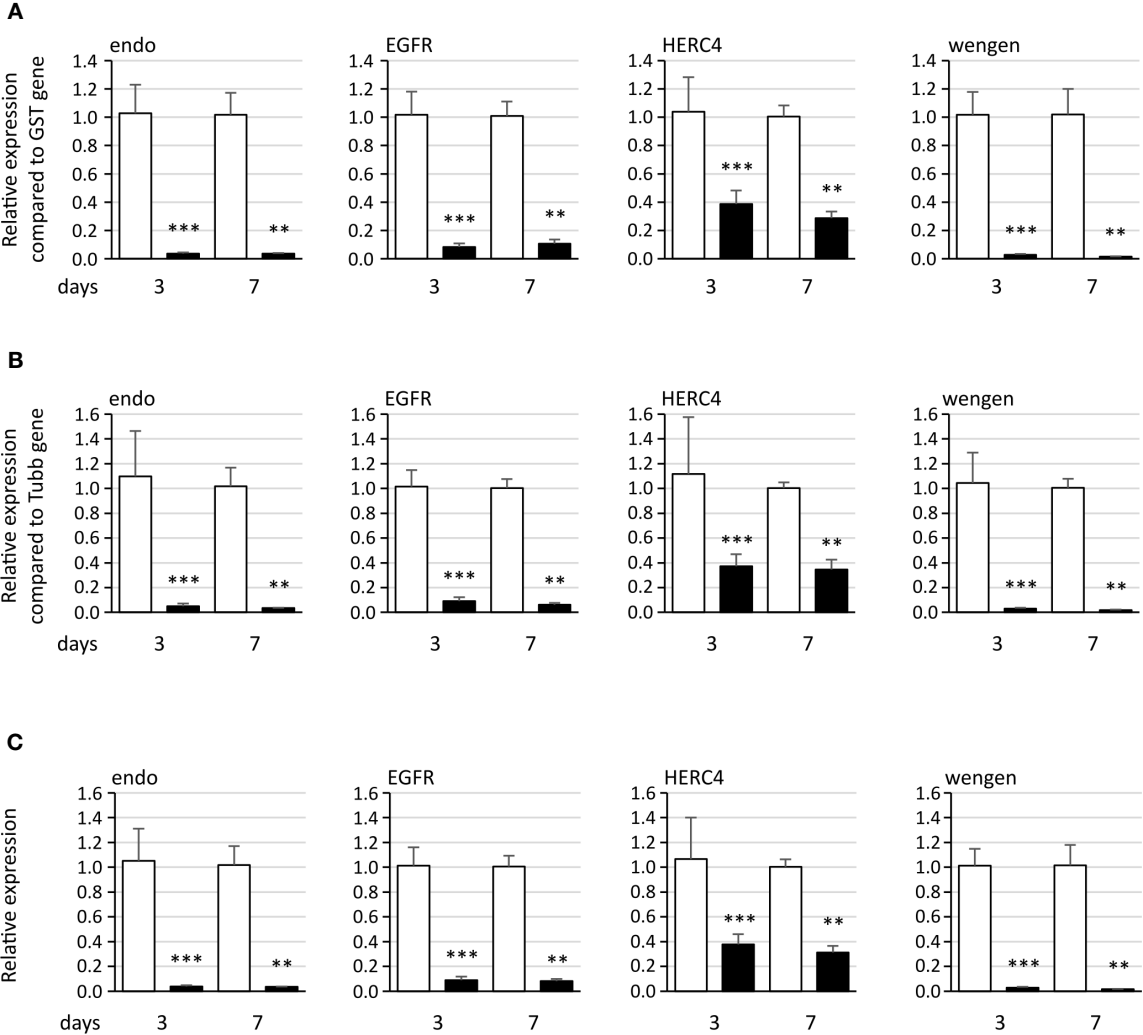
Expression of the four genes endoplasmin (endo), epidermal growth factor receptor (EGFR), HERC4 E3 ubiquitin-protein ligase (HERC4), and tumor necrosis factor receptor superfamily member wengen (wengen) in Euva cells with regard to the reference gene glutathione S-transferase **(A)**, tubulin β **(B)**, and both genes **(C)**. White boxes correspond to Euva cells transfected with GFP dsRNA, and black boxes correspond to Euva cells transfected with dsRNA targeting the gene indicated above the graphs. ** indicates a significant difference with p < 0.01 and *** with p < 0.001 under the Kruskal–Wallis rank-sum test of the R commander package of R software version 4.0.3.

RNA interference (RNAi) was used in the Euva cells to inhibit the production of the candidate proteins interacting with VmpA-His_6_. Quantification of the mRNA of the four first candidates, *i.e.*, endo, EGFR, HERC4, and wengen, was performed 3 and 7 days after insect cell transfection with dsRNA. The dsRNA control corresponded to the GFP gene. Total RNAs were extracted, and real-time RT-PCR was used to quantify the effect of dsRNAs on the expression of these four genes. Three relative expression levels were calculated using the glutathione S-transferase (GST) and tubulin β (tubβ) genes as references. A significant inhibition was observed 3 and 7 days post-transfection with dsRNA, irrespective of the gene considered (**Figure 3**). The reduction of specific transcripts varied between 3- and 68-fold depending on the gene and the time post transfection. No difference in inhibition rates was observed when we referred to the two reference genes, so only the GST reference gene was kept for the further experiments. No differences were observed between 3 and 7 days whatever the gene considered except for the wengen gene expression for which a slight decrease was observed at 7 days (*p* = 0.03 under the Kruskal–Wallis rank-sum test of the R commander package of R software version 4.0.3). Therefore, extraction of mRNAs and further experiments were performed 3 days post-transfection that should be adequate for turnover of the target protein as mentioned in Clemens et al. (Clemens et al., 2000).

**Figure 4:**
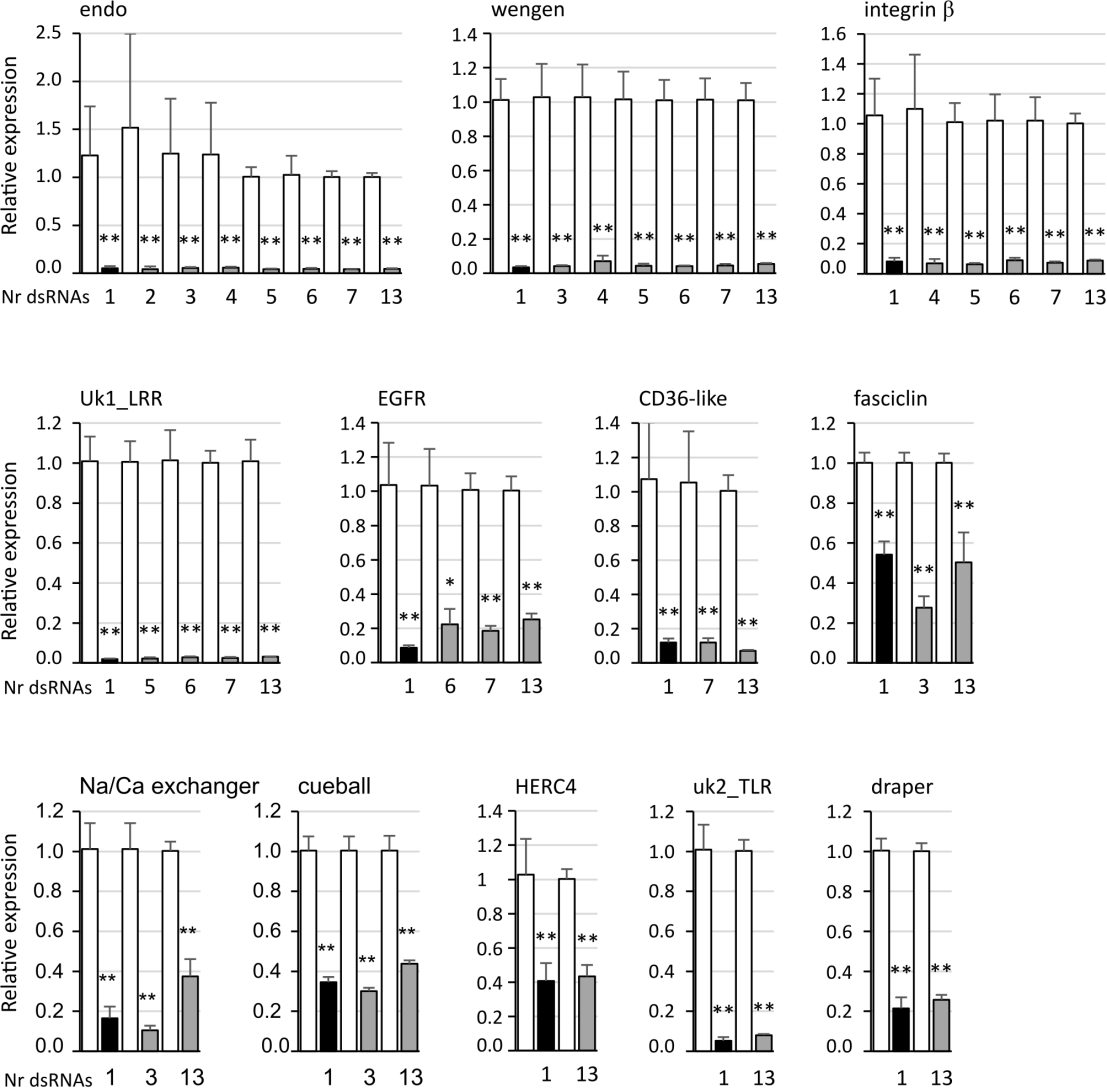
Expression of the candidate genes endoplasmin (endo), tumor necrosis factor receptor super family member wengen (wengen), integrin β protein containing LRR domains (uk1_LRR), epidermal growth factor receptor (EGFR), lysosome membrane protein 2 (CD36-like), transforming growth factor-beta-induced protein ig-h3-like (fasciclin), sodium/calcium exchanger 3-like isoform X1 (Na/Ca exchanger), protein cueball isoform X1 (cueball), HERC4 E3 ubiquitin-protein ligase (HERC4), leucine-rich repeat-containing protein 15-like (uk2_TLR), and protein draper isoform X1 (draper) in Euva cells with regard to the reference gene glutathione S-transferase. White boxes correspond to Euva cells transfected with GFP dsRNA, black boxes with dsRNA targeting the gene indicated above the graphs, and gray boxes with several dsRNA including the gene studied, the number of which is indicated under the graph. * indicates a significant difference with p < 0.05 and ** with p < 0.01 under the Kruskal–Wallis rank-sum test of the R commander package of R software version 4.0.3 with regard to the GFP dsRNA control.

When we tested the nine other genes, we found that the expression rate of the gene uk3 was too low for the RNAi assay to be performed (Ct values of 34–35). For the remaining eight genes, namely, integrin β, uk1_LRR, CD36-like, fasciclin, Na/Ca exchanger, cueball, uk2_TLR, and draper, a statistically significant inhibition of gene expression was achieved (**Figure 4**, black bars) with fold change values ranging from 2.03 for the fasciclin gene to 51.4 for the uk1_LRR gene.

### 3.4 Simultaneous multiplexing RNAi on Euva cells

The adhesin VmpA potentially interacts with several insect receptors, as multiple bands were observed after far-western blot with VmpA-His_6_ (**Figure 2**). To maximize the chances to inhibit different VmpA receptor(s) at once, we performed assays in which several genes were inhibited simultaneously with dsRNA targeting the different genes selected. For all the 12 genes, significant differences were observed with the control condition, *i.e.*, GFP dsRNA, whatever the number of genes inhibited simultaneously including the targeted gene (**Figure 4**, gray bars). For the genes endo, wengen, integrin β, uk1_LRR, CD36-like, fasciclin, cueball, HERC4, uk2_TLR, and draper, no difference was observed between the inhibition with dsRNA targeting the gene studied alone and the inhibition induced with up to 13 several different dsRNA, including the targeted gene studied. The dsRNAs designed on uk3 sequences was also included in the multiplex silencing although its inhibition in transfected cells was not assessed. For the genes EGFR and Na/Ca exchanger, a lower gene expression inhibition efficiency was observed when 12 other genes were simultaneously inhibited with the gene studied compared to the single inhibition of the EGFR and Na/Ca exchanger genes.

**Figure 5:**
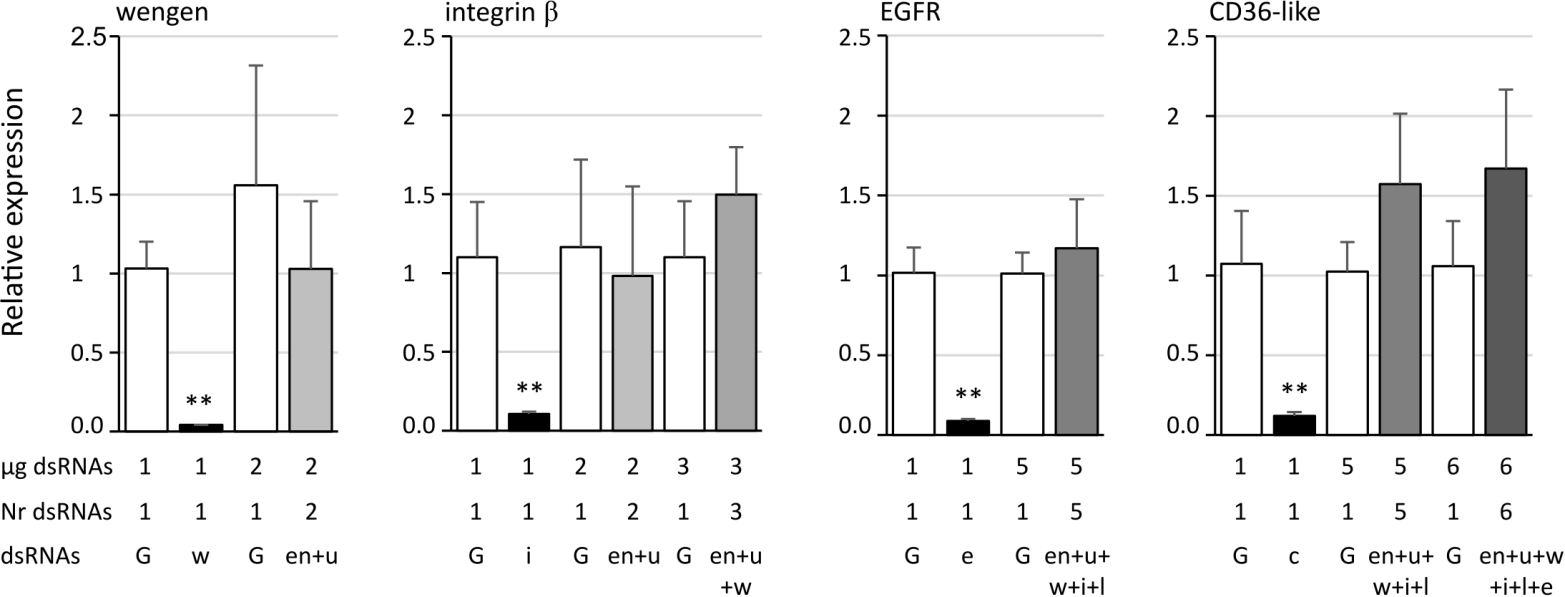
Expression of the candidate genes tumor necrosis factor receptor superfamily member wengen (wengen), integrin β, epidermal growth factor receptor (EGFR), and lysosome membrane protein 2 (CD36-like) in Euva cells with regard to the reference gene glutathione S-transferase. White boxes correspond to Euva cells transfected with GFP dsRNA (G) at the quantities indicated under the graph (µg dsRNA), black boxes with dsRNA targeting the gene studied and indicated above the graphs, and gray boxes with several dsRNA without dsRNA targeting the gene studied, the number of which is indicated under the graph (Nr dsRNA). dsRNAs indicates the targeted genes endoplasmin (en), uk3 (u), wengen (w), integrin β (i), unk1_LRR (l), CD36-like (c), and EGFR (e). ** indicates a significant difference with p < 0.01 under the Kruskal–Wallis rank-sum test of the R commander package of R software version 4.0.3 with regard to the GFP dsRNA control.

We tested the effect of off target on the expression of four genes when two to six other genes were simultaneously inhibited. The results show that no significant difference was observed when dsRNA targeted genes other than the studied one compared to GFP dsRNA (**Figure 5**).

It must be noticed that the phenotype of transfected cells observed with light microscope and their ability to achieve confluence was not impacted by the silencing of any of the candidate genes nor when the 13 candidate genes were inhibited simultaneously (**Supplementary Figures S1**, **S2**).

### 3.5 Adhesion assays to Euva cells of VmpA-coated beads

**Figure 6:**
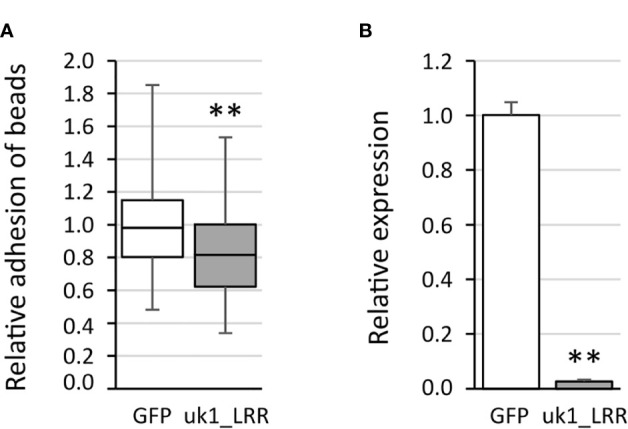
Adhesion of the VmpA-His_6_-coated beads to the Euva-11 cells in the presence of dsRNA of the uk1_LRR gene and GFP as control. **(A)** Adhesion of VmpA-His_6_-coated beads to Euva-11 cells 3 days after the cells were transfected with GFP dsRNA (white) or uk1_LRR dsRNA (gray). ** indicates a significant difference with *p* = 0.003671 for uk1_LRR under the Kruskal–Wallis rank-sum test. **(B)** Control of RNAi efficiency into Euva-11 cells transfected at the same time as cells incubated with beads in **(A)** The expression of the uk1_LRR gene was measured in Euva-11 cells with regard to the reference gene glutathione S-transferase. White box corresponds to Euva-11 cells transfected with GFP dsRNA and gray box with uk1_LRR dsRNA. ** indicates a significant difference with *p* = 0.0004912 under the Kruskal–Wallis rank-sum test of the R commander package of R software version 4.0.3 with regard to the GFP dsRNA control.

The VmpA-His_6_-bead adhesion assays were first performed 3 days after transfection using Euva-11 cells transfected with dsRNA targeting one gene. On average, 51 ( ± 5) Euva-11 cells and 200 ( ± 50) beads were observed per field. There were 20 fields photographed per condition, and three independent experiments were performed and pooled. The results show that, whatever the gene inhibited, no difference of bead adhesion was observed between the cells transfected with the dsRNA targeting candidate genes and GFP dsRNA (**Supplementary Figure S3A**) except for the gene uk1_LRR (**Figure 6A**). For the gene uk1_LRR, the three repetitions were very homogeneous, *i.e.*, 0.89 ± 0.21, 0.87 ± 0.25, and 0.87 ± 0.28 of relative adhesion of beads with medians of 0.90, 0.82, and 0.75, respectively. At the same time as adhesion assays, the inhibition of the targeted genes was checked. Results show that for each gene, a significant decrease of the mRNA was measured (**Figure 6B**, **Supplementary Figure S3B**). The strongest inhibitions were observed for the genes uk1_LRR and wengen with 38.5-fold and 33-fold change of mRNA quantities respectively, and the weakest inhibition was observed for the gene fasciclin with twofold change.

As inhibition of the 13 genes simultaneously by RNAi did not show difference with the inhibition of a single gene, we thus inhibited the 13 genes simultaneously before to incubate the transfected cells with VmpA-His_6_-coated beads. Unfortunately, no statistically difference in adhesion of VmpA-His_6_-coated beads has been observed on cells where the expression of the 13 candidates was inhibited at once.

### 3.6 Characterization of the uk1_LRR gene

**Figure 7:**
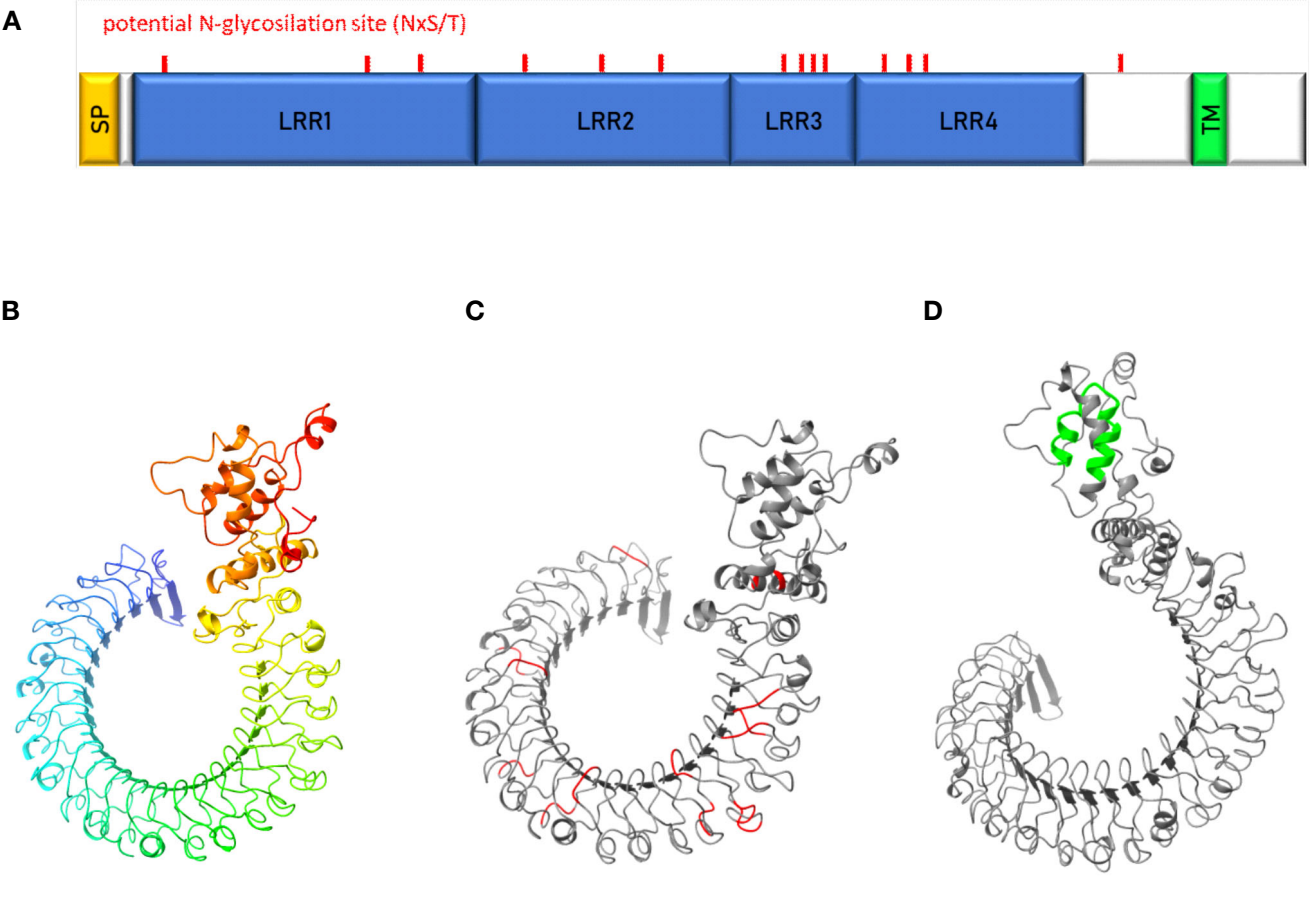
Characterization of uk1_LRR protein. **(A)** Schematic representation of uk1_LRR protein where the signal peptide (SP) predicted by InterPro and TMHMM is colored in yellow, the LRR domains predicted by InterPro in blue, and the transmembrane domain predicted by TMHMM (TM) in green. Predicted protein structures by Robetta (Baker Lab) were visualized with the software Chimera X (v.1.5) without signal peptide **(B)**, with the potential N-glycosylation sites (aminoacidic motif « NxS/T ») highlighted in red **(C)** and the transmembrane domain highlighted in green **(D)**.

The uk1_LRR amino acidic and nucleotide sequences were investigated through a BLAST search, revealing several homologs in other organisms present in the databases (**Table S3**). Uk1_LRR was predicted by TMHMM2.0 to be a transmembrane protein anchored in the plasma membrane by a single 22–23-amino acids-long transmembrane segment located in the C-terminal part (**Figures 7A, D**). Most of the proteins were predicted to be exposed to the cell surface. The uk1_LRR protein contains four leucine-rich-repeat (LRR) domains of 219, 163, 80, and 146 aa (from N-ter to C-ter sequence) classified in the ribonuclease inhibitor-like group. Furthermore, consistent with sequence prediction indicating LRR domains, structural prediction (Robetta, Baker Lab) revealed a horseshoe-shaped molecule consisting of parallel β-strands on the inner concave side and helical elements on the outer convex sites (**Figure 7B**). There were 14 potential N-glycosylation sites (NxST motif) found in the uk1_LRR amino acid sequence and are localized on the protein predicted structure in **Figure 7C**.

No paralogous gene was found in the *E. variegatus* transcriptome. However, it has been possible to identify an ortholog gene in *S. titanus* (Transcriptome Shotgun Assembly ref. “Scaphoideus titanus taxid:376741”), the natural vector of flavescence dorée phytoplasmas, with an amino acid identity of 90.6% (Clustal 2.1). *E. variegatus* uk1_LRR consists of 781 amino acids while the *S. titanus* sequence is 779 amino acids long. Potential N-glycosylation sites were conserved between the two proteins, both in number (14) and in position, with a shift that reflects the difference in protein sequence length.

### 3.7 Assessment of uk1_LRR expression in *E. variegatus*

**Figure 8:**
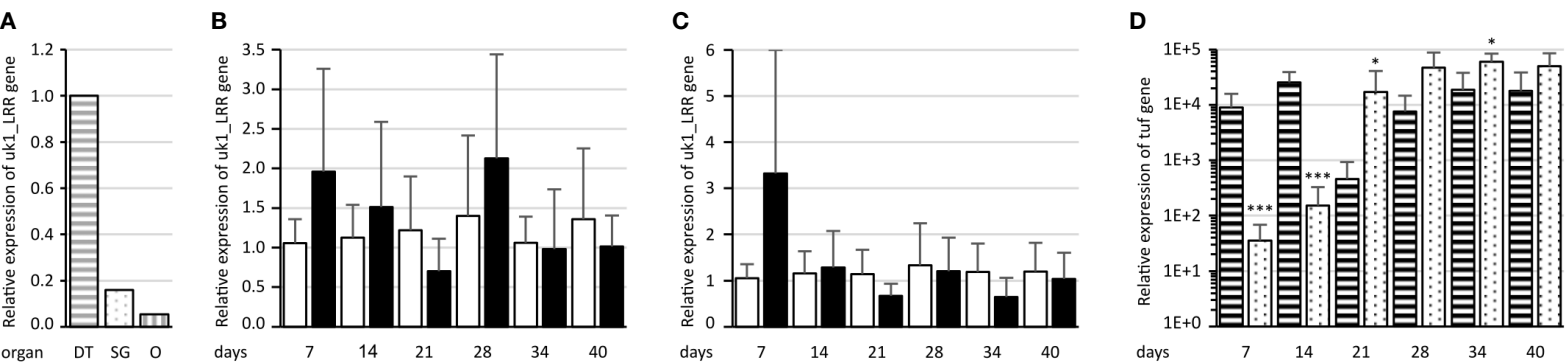
Assessment of uk1_LRR expression in *E*. *variegatus*. **(A)** Relative expression of uk1_LRR in the *E*. *variegatus* digestive tube (gray horizontal stripes) and salivary glands (gray dots) of male and female insects, and ovary and eggs (gray vertical stripes). **(B)** Relative expression of uk1_LRR of *E*. *variegatus* with regard to the reference gene glutathione S-transferase in abdomens or **(C)** in heads at different time points on non-infected plants (white bars) or on FD92 phytoplasma-infected plants (black bars). **(D)** Relative expression of the phytoplasma tuf gene at different time points in abdomens (horizontal stripes) and heads (dots) of *E*. *variegatus*. * indicates a significant difference with p < 0.05 and ***p < 0.001 (under the Kruskal–Wallis rank-sum test of the R commander package of R software version 4.0.3) in tuf expression between abdomens and heads at each time point.

To assess uk1_LRR expression in the different organs of the insect vector, real-time RT-PCR was performed on salivary glands, digestive tubes, and ovaries with eggs dissected from healthy *E. variegatus*. The organ showing the highest uk1_LRR expression values was the digestive tube (**Figure 8A**). The gene was expressed 6 and 18 times less in the salivary glands and ovaries, respectively, when compared to digestive tubes.

uk1_LRR expression was then assessed on synchronized *E. variegatus* adults up to 40 days after feeding onto FD92 phytoplasma-infected broad beans or onto healthy plants as control, both in abdomens (**Figure 8B**) and in heads containing the salivary glands (**Figure 8C**). Even though no statistically significant differences were found in expression of uk1_LRR between healthy and FD92-infected insects, a trend is clearly visible. At 7 days post acquisition, the uk1_LRR gene was more expressed in FD92-infected insects, both in the heads and in the abdomens, than in non-infected insects. While the uk1_LRR gene expression remained stable in the insect that fed on non-infected plants, the uk1_LRR mRNA decreased over time in the insects fed on phytoplasma-infected plants. An increase in abdomens and heads was observed 28 days post acquisition, before uk1_LRR mRNA expression decreased.

As far as phytoplasma tuf mRNA relative quantities are concerned, both heads and abdomens were found to be positive to phytoplasma detection since the first sampling date, 7 days after the beginning of acquisition (**Figure 8D**). At 7 and 14 days, quantities of tuf mRNA in the abdomens were significantly higher than in the heads, then titers were significantly higher in the heads than in the abdomens. The quantities of tuf mRNA were nearly stable in the abdomens over the latency whereas they increased over time in the heads.

## 4 Discussion

Mechanisms driving the colonization of insect vector cells by phytoplasma are poorly deciphered. The first phytoplasma protein able to interact with protein of insect vector was the Antigenic Membrane Protein AMP. It was demonstrated that AMP of “*Candidatus* phytoplasma asteris” was able to interact with the cellular microfilament and more specifically with the actin of vectoring leafhopper species but not with actin of non-vectoring leafhopper species (Suzuki et al., 2006). In addition, the amp gene and its ortholog stamp in “*Ca*. P. solani” were shown to be submitted to heavy positive selection indicating a possible adaptation process acting on this gene (Kakizawa et al., 2006; Fabre et al., 2011). However, the interaction of AMP with actin is certainly involved in the intracellular trafficking of phytoplasmas once they are internalized into the insect vector cells. Prior to this step, phytoplasma must interact with the surface proteins of the insect cells. Our recent work demonstrated that VmpA and VmpB proteins of group 16SrV-C and V-D phytoplasmas are highly variable and that their variability correlates with the specific transmission of Vmp variants by leafhopper of the subfamily *Deltocephalinae* (Malembic-Maher et al., 2020). We could previously demonstrate that VmpA acts as an adhesin able to bind cells of its insect vector in a lectin-like manner (Arricau-Bouvery et al., 2018; Arricau-Bouvery et al., 2021). After this initial step of adhesion, flavescence dorée phytoplasma enters insect cells by a clathrin-mediated endocytosis allowing infection of its insect vector (Arricau-Bouvery et al., 2023). We currently hypothesize that leafhopper species able to acquire FD phytoplasma have a glycoprotein set different to that of leafhopper species unable to achieve acquisition. Variations in the cortege of glycoproteins can lead to variations in the binding of lectins at the surface of hemipteran insect cells. For instance, an enhanced binding of the mannose-specific *Galanthus nivalis* lectin GNA to the gut glycoproteins of the rice planthopper *Nilaparvata lugens* by comparison to that of the rice leafhopper *Nephotettix cincticeps* is associated to a higher toxicity of GNA to *N. lugens* (Foissac et al., 2000). To identify the glycoprotein receptors for VmpA, we enrich Euva cell membrane protein fractions into proteins able to interact with VmpA. However, the number of proteins identified through mass spectrometry was large. We decided to focus our attention to the protein candidates predicted to be surface membrane proteins with a potential site for N-glycosylation. In order to validate their role in VmpA binding, we measured the binding of VmpA to Euva cells in which the expression of the corresponding gene was silenced.

RNAi is an efficient selective silencer of target genes, but it is not absolutely specific. Three main off-target effects due to siRNA delivery in an organism can be described and are reviewed by Jackson and Linsley (Jackson and Linsley, 2010). The first one, which is expected to not have influence in our study, is an inflammatory response against the siRNAs and/or the transfectant used to deliver dsRNAs in mammals. The second off-target effect is linked to the siRNA-induced sequence-dependent regulation of unintentional transcripts through partial sequence complementary to their 3′UTRs. Checking the effect on the other target genes showed that no off-target effect was observed in our case. However, even though no partial sequence homology was found in the *E. variegatus* transcriptome for the genes we tested, we cannot exclude that the expression of other genes could have been affected. A study showed that pooling 10 synthetic siRNAs targeting the same mRNA reduced the number and magnitude of off-target silencing compared to the use of a single siRNA (Kittler et al., 2007). In our case, we used long dsRNA instead of siRNA. The dsRNAs are taken by the RNAi machinery to produce several siRNAs targeting the same mRNA and could induce a reduction of off-target effects. The third off-target effect is due to a saturation of the endogenous RNAi machinery by exogenous siRNAs. In our case, when we compared the efficiency of the RNAi using one dsRNA (1 µg of dsRNAs) or up to 13 different dsRNA (13 µg of dsRNAs), we did not find any differences for 10 over 12 genes targeted suggesting that the quantity of dsRNAs used did not saturate the RNAi machinery. Usually, one gene at a time is knockdown by RNAi using either long dsRNA or multiple siRNAs targeting one gene and introduced by transfection into insect cells or vertebrate cells, respectively. We did not find examples of cultured cells transfected with multiple long dsRNA targeting several genes. However, an example of three genes silenced using Y-shaped siRNAs was described in a bone metastatic prostate cancer cell line (Kozuch et al., 2018). RNAi targeting several genes has been described in insects that were injected with or ingested dsRNA. For example, when two genes were targeted with two dsRNAs injected into the planthopper *N. lugens* or with two dsRNAs or a concatemer ingested by the whitefly *Bemisia tabaci*, knockdown of genes were similar to that observed with injection or ingestion of individual dsRNA. Use of concatemer showed an increase of gene knockdown when compared to mixed dsRNAs (Mao et al., 2021; Jain et al., 2022).

Using VmpA-coated beads instead of living phytoplasmas allowed us to avoid problems due to the concentration and the viability of phytoplasmas recovered from infected insects or infected plants. Moreover, this simplified system allows us to study the role of the sole VmpA in the interaction with the insect cells without the masking effect of other Vmps or membrane factors. This strategy, applied to 12 candidates, revealed the possible implication of *E. variegatus* uk1_LRR protein in the adhesion to the vector cell mediated by phytoplasma strain FD92 VmpA. Indeed, the inhibition of the adhesion of VmpA-coated beads to Euva cells in culture was only partially achieved. The amount of uk1_LRR transcripts, as all of the gene candidates tested, was successfully decreased through RNAi but not completely knockout.

We have only few indications about the uk1_LRR function. Proteins with such LRR structures are frequently implicated in protein–protein interactions and have a variety of functions including immune response, apoptosis, autophagy, and neuronal development (Kobe and Kajava, 2001; Matsushima et al., 2019). The uk1_LRR protein of *E*. *variegatus* contains LRR domains classified in the ribonuclease inhibitor (RI)-like group. However, uk1_LRR does not show sequence homology with RI and is predicted to be exposed to the surface cell whereas the RI protein is cytosolic (Dickson et al., 2005). It is then highly probable that the uk1_LRR protein has a different function from RI. The other proteins sharing homologies with the uk1_LRR protein also contain the LRR domain classified in the RI-like group. However, the diversity of the proteins found, *i.e.*, artichoke-like protein, Toll pathway protein, or Toll-like receptor Tollo, as examples, only share their extracellular position and LRR domains. Several proteins matching amino acidic sequence of uk1_LRR in the homology search were annotated as carboxypeptidase N subunit-like. However, the study of uk1_LRR sequence revealed the absence of carboxypeptidase domains. Therefore, the function of uk1_LRR remains to be identified.

We found that uk1_LRR was expressed in both phytoplasma-infected and uninfected insect vectors and was more or less stable over time during insect colonization by the phytoplasma. In the early stages of vector infection, right after ingestion of phytoplasma, the expression of uk1_LRR had a tendency to increase in infected insects when compared to same-age adults fed on healthy plants. Interestingly, a similar increase in expression was found in infected insect abdomens at 28 days of latency, when insects acquire again phytoplasmas from the faba beans that became systemically infected. Indeed, at this timepoint, the phytoplasma titer measured in insect abdomens increased, suggesting that the expression level of uk1_LRR follows the *de novo* ingestion of phytoplasmas. Furthermore, the evaluation of uk1_LRR expression in different insect tissues revealed that the digestive tubes were characterized by the highest level of transcripts when compared to salivary glands and ovaries and eggs. Taking consideration of these altogether, uk1_LRR could be implicated in the binding with VmpA in the early stages of insect infection following phytoplasma ingestion, *i.e.*, at the surface of intestine epithelial cells.

## Data availability statement

The mass spectrometry proteomics data have been deposited to the ProteomeXchange Consortium via the PRIDE [1] partner repository with the dataset identifier PXD046447.

## Ethics statement

The manuscript presents research on animals that do not require ethical approval for their study.

## Author contributions

FC: Conceptualization, Formal Analysis, Investigation, Methodology, Writing – original draft, Writing – review & editing. SD: Conceptualization, Formal Analysis, Investigation, Methodology, Writing – review & editing. M-PD: Conceptualization, Formal Analysis, Investigation, Methodology, Writing – review & editing. SC: Formal Analysis, Investigation, Writing – review & editing. SM-M: Writing – review & editing. XF: Conceptualization, Formal Analysis, Funding acquisition, Investigation, Project administration, Supervision, Writing – original draft, Writing – review & editing. NA-B: Conceptualization, Formal Analysis, Funding acquisition, Investigation, Methodology, Project administration, Supervision, Writing – original draft, Writing – review & editing.
