# Supplementary material for: A knockdown gene approach identifies an insect vector membrane protein with leucin-rich repeats as one of the receptors for the VmpA adhesin of flavescence dorée phytoplasma
Source: Front Cell Infect Microbiol. 2023 Nov 6;13:1289100. doi: 10.3389/fcimb.2023.1289100 (PMC10662966; doi:10.3389/fcimb.2023.1289100)
Supplement: Supplementary file 1 [file DataSheet_1.pdf]

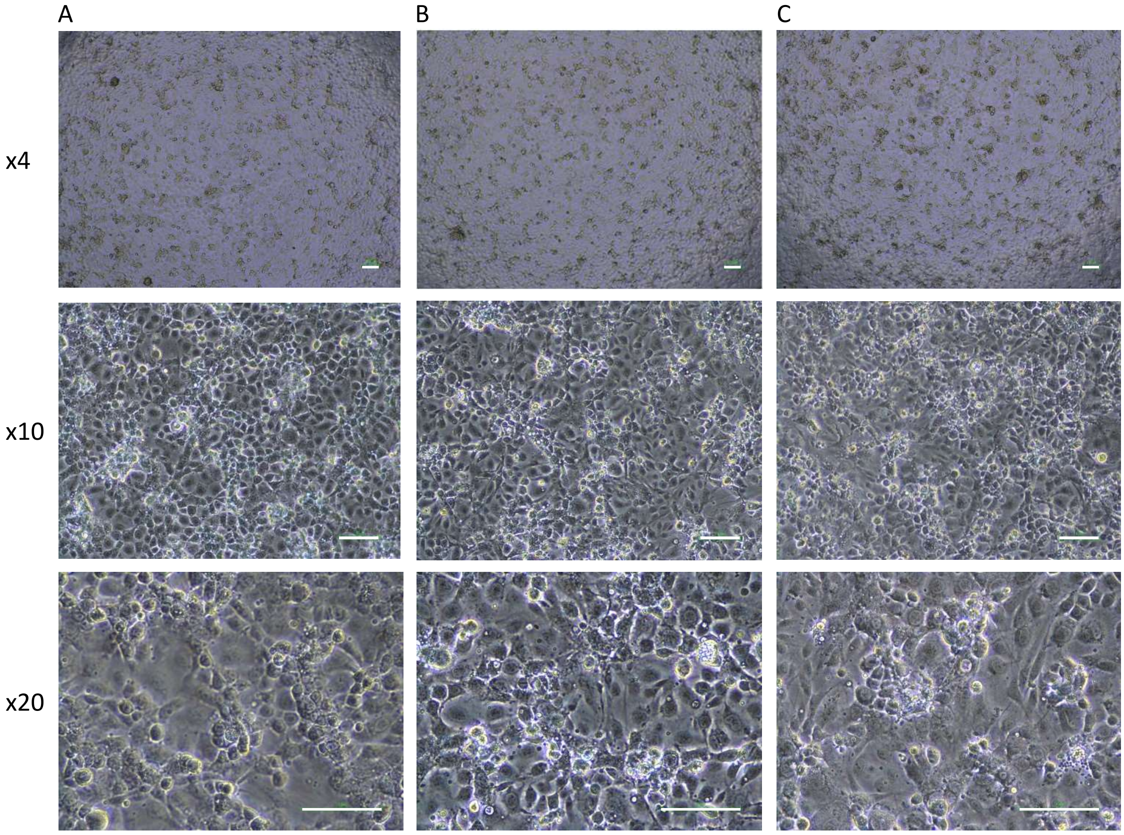

Supplementary Figure 1. Euva11 cells observed at the inversed microscope 3 days post transfection. (A) Control condition in which the Euva-11 cells were cultivated in culture medium without transfection for 3 days, (B) after transfection with GFP dsRNA and (C) after transfection with uk1-LRR dsRNA. The magnification of observations is shown to the left of the panels. Scale bar 100 µm.
