# Supplementary material for: A knockdown gene approach identifies an insect vector membrane protein with leucin-rich repeats as one of the receptors for the VmpA adhesin of flavescence dorée phytoplasma
Source: Front Cell Infect Microbiol. 2023 Nov 6;13:1289100. doi: 10.3389/fcimb.2023.1289100 (PMC10662966; doi:10.3389/fcimb.2023.1289100)
Supplement: Supplementary file 2 [file DataSheet_2.pdf]

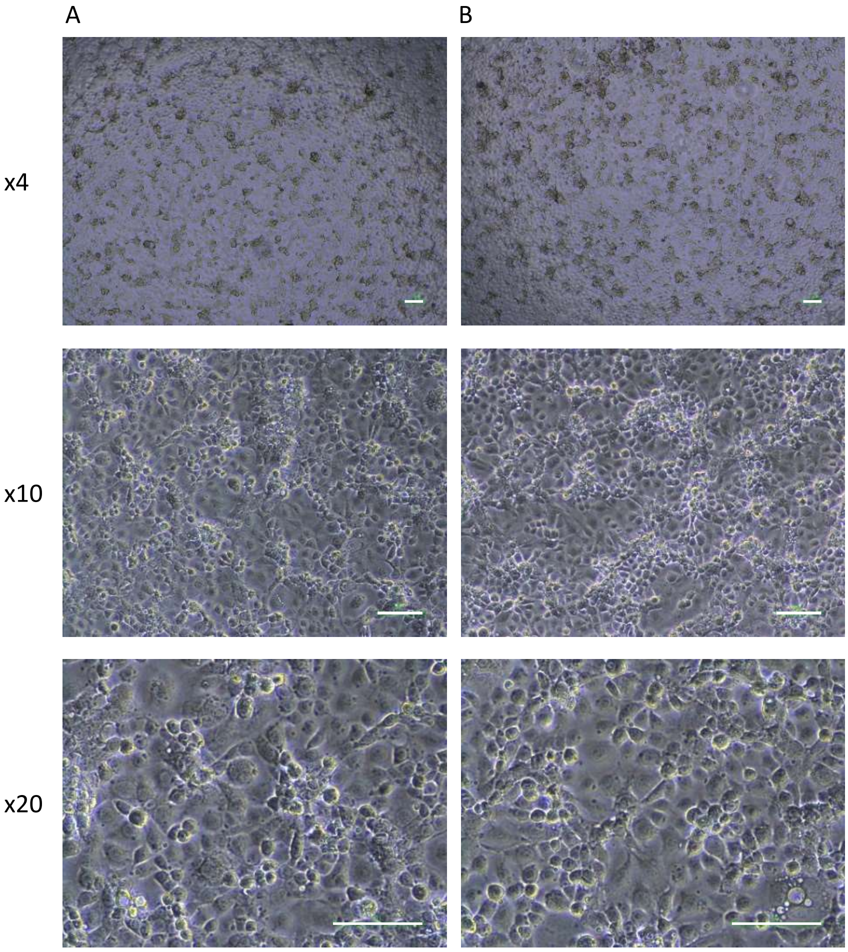

Supplementary Figure 2. Euva11 cell line observed at the inversed microscope at 3 days post transfection. Euva11 cells transfected with 13 µg of dsRNAGFP (A) and 1µg of each of the 13 candidates selected for screening (B). The magnification of observations is shown to the left of the panels. Scale bar 100 µm.
