# Supplementary material for: A knockdown gene approach identifies an insect vector membrane protein with leucin-rich repeats as one of the receptors for the VmpA adhesin of flavescence dorée phytoplasma
Source: Front Cell Infect Microbiol. 2023 Nov 6;13:1289100. doi: 10.3389/fcimb.2023.1289100 (PMC10662966; doi:10.3389/fcimb.2023.1289100)
Supplement: Supplementary file 3 [file DataSheet_3.pdf]

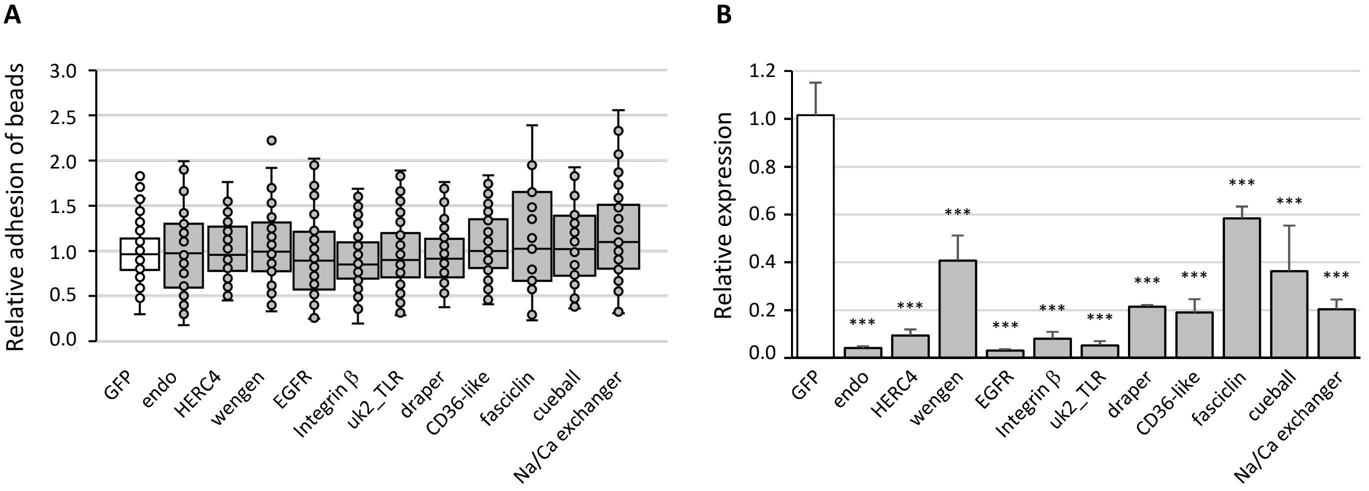

Supplementary Figure 3. Adhesion of the VmpA-His$_6$-coated beads to the Euva-11 cells in presence of dsRNA of candidate genes and GFP as control. A, adhesion of VmpA-His$_6$-coated beads to Euva-11 cells three days after the cells were transfected with GFP dsRNA (white) or dsRNA of candidate genes (grey) the name of which is indicated under the graph. B, control of RNAi efficiency into Euva-11 cells transfected at the same time as cells incubated with beads in A. The expression of the candidate genes in Euva-11 cells was measured regards to the reference gene glutathione S-transferase. White box corresponds to Euva-11 cells transfected with GFP dsRNA and grey boxes with dsRNA targeting the gene indicated above the graphs. *** indicates a significant difference with $p<0.001$, under the Kruskal-Wallis rank sum test of the R commander package of R software version 4.0.3 regards to the GFP dsRNA control.
