# Supplementary material for: A knockdown gene approach identifies an insect vector membrane protein with leucin-rich repeats as one of the receptors for the VmpA adhesin of flavescence dorée phytoplasma
Source: Front Cell Infect Microbiol. 2023 Nov 6;13:1289100. doi: 10.3389/fcimb.2023.1289100 (PMC10662966; doi:10.3389/fcimb.2023.1289100)
Supplement: Supplementary file 4 [file DataSheet_4.pdf]

A

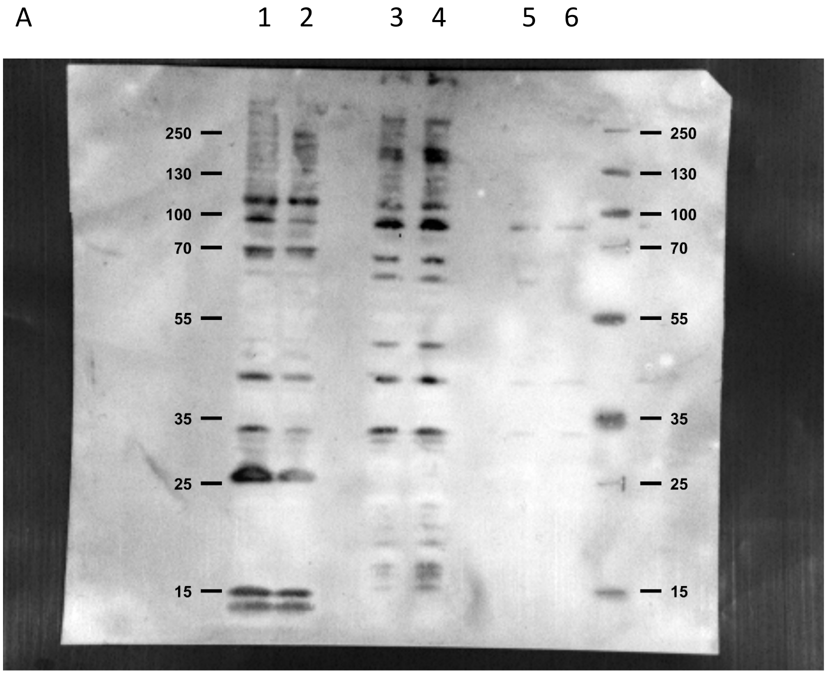

B

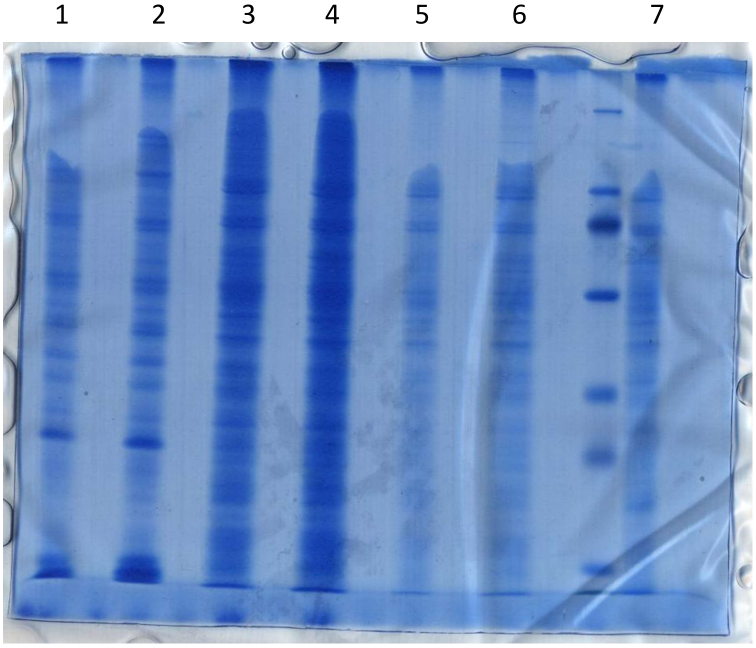

Supplementary Figure 4: Interaction of VmpA-His$_6$ with Euva cell proteins in vitro (scan of the complete far western blot and Coomassie blue gel in Figure 2). (A) Euva proteins transferred to nitrocellulose membrane were incubated with recombinant VmpA-His$_6$. (B) Euva proteins coloured with Coomassie blue. Euva-12 (1) and Euva-11 (2) proteins present in the pellet (insoluble fraction). Euva-12 (3) and Euva-11 (4) proteins soluble in Rx-T-DOC buffer. Euva-12 (5) and Euva-11 (6) proteins soluble in the Rx buffer. Euva-6 proteins extracted with Rx buffer (7).
