# Supplementary material for: A knockdown gene approach identifies an insect vector membrane protein with leucin-rich repeats as one of the receptors for the VmpA adhesin of flavescence dorée phytoplasma
Source: Front Cell Infect Microbiol. 2023 Nov 6;13:1289100. doi: 10.3389/fcimb.2023.1289100 (PMC10662966; doi:10.3389/fcimb.2023.1289100)
Supplement: Supplementary file 5 [file DataSheet_5.pdf]

Supplementary Table 1. List of primers and plasmids used in this study. T7 promoter sequence is in bold.

Target gene	Primer names	5'-3' sequence	Product size (n)	Application	PCR efficiency (%)	Plasmid used	Plasmid resulting
endoplasmin	c_endoplasmine_F	cccATGGGGAGAAAACATTCTCACC	2358	cloning	-	pGEMT-Easy	pGEMT-Easy_endoplasmine
	c_endoplasmine_R	CGGTTACAGTTCGTCGTGTTCC					
	db_endoEv_T7p_F	**TAATACGACTCACTATAGGGG**GAGGACAGCGAGTACCATGA	495	dsRNA synthesis	-		
	db_endoEv_T7p_R	**TAATACGACTCACTATAGGGG**TACCTTGGACGACTGGAACC					
	q_endo_F1	CATTACGGACACAGGCATCG	224	RT-PCR	2.033		
	q_endo_R1	AGACCAGCTGTTTGTCCTCA					
EGFR	c_EGFR_F3	GACTGCACCGTTATCGAG	1137	cloning	-	pMINIT_2.0	pMINIT_2.0_EGFR
	c_EGFR_R4	ACCGTAGCAGCCTCTGCAC					
	db_EGFR_T7p_F	**TAATACGACTCACTATAGGGG**TGCTCAGGGAGTGGACTGGAAG	417	dsRNA synthesis	-		
	db_EGFR_T7p_R	**TAATACGACTCACTATAGGGG**CCCTGGCAGCCGTACACACAG					
	c_EGFR_F3	GACTGCACCGTTATCGAG	246	RT-PCR	2.01		
	q_EGFR_R3	GTATTCCGTCAATGTTCTTCCT					
HERC4	c_HERC4_Ev_F	CAACGCCAAGAACTGTGACAAG	916	cloning	-	pMINIT_2.0	pMINIT_2.0_HERC4
	c_HERC4_Ev_R	TCCGAGCAGCTTCTTGTACAG					
	db_HERC4_T7p_F	**TAATACGACTCACTATAGGGG**AGATCAAGCCCTACAGATGC	431	dsRNA synthesis	-		
	db_HERC4_T7p_R	**TAATACGACTCACTATAGGGG**GGAATGGCAGCGCGATGATG					
	c_HERC4_Ev_F	CAACGCCAAGAACTGTGACAAG	221	RT-PCR	2.04		
	q_HERC4_R	CCGTTAGCATCCCCTCGTAC					
wengen	c_wng_F	ATGGGGATAAATACAAAGAAGG	1516	cloning	-	pMINIT_2.0	pMINIT_2.0_TNFwng
	c_wng_R	CTTAGCTAAGCGGTCTTTTCTG					
	db_wng_T7p_F	**TAATACGACTCACTATAGGGG**AGGATTAGCACCACCTGTTG	404	dsRNA synthesis	-		
	db_wng_T7p_R	**TAATACGACTCACTATAGGGG**CGGGGTTCAATAGATGCGAC					
	q_wng_F	CGGTCTGCAGTACTGGAGC	223	RT-PCR	1.903		
	q_wng_R	CTTCTCGGCTGACACGTCGC					
uk1_LRR	LRR_15247_cF	ATGGGCAGTGCGAGTGCAG	2350	cloning	-	pMINIT_2.0	pMINIT_2.0_LRR
	LRR_15247_cR	GCTGTCATTTACGATGTTTCACC					
	db_LRR_15247_T7p_F	**TAATACGACTCACTATAGGGG**GACGACTGCGACTGCCACTAC	483	dsRNA synthesis	-		
	db_LRR_15247_T7p_R	**TAATACGACTCACTATAGGGG**CGGGTTGCCTGACAGATCTAG					
	LRR_15247_qF	CTTAGCAGCCCAGAGAACG	265	RT-PCR	1.936		
	LRR_15247_cR	GCTGTCATTTACGATGTTTCACC					
uk2_TLR	TLR_14488_cF	ATGAAGGTCGCTGTAATACTCTC	2170	cloning	-	pMINIT_2.0	pMINIT_2.0_TLR
	TLR_14488_cR	GGAATGTTTACACGACATCATCA					
	TLR_144888_T7p_F	**TAATACGACTCACTATAGGGG**GCAGATATCCATGAGTAGTTCG	610	dsRNA synthesis	-		
	TLR_144888_T7p_R	**TAATACGACTCACTATAGGGG**CTGTGGCCTGTCTAACATTTCC					
	TLR_14488_cF	ATGAAGGTCGCTGTAATACTCTC	239	RT-PCR	1.942		
	TLR_14488_qR	GCTTTCTCCAATCCGATGG					
CD36-like	CD36_cF	ATGCAGAACAAACGGGGG	1623	cloning	-	pMINIT_2.0	pMINIT_2.0_CD36
	CD36_cR	TTACGTTGTGTGTTCGTCG					
	db_CD36_T7pF	**TAATACGACTCACTATAGGGG**GAGTTCGGACTGATGTATGG	549	dsRNA synthesis	-		
	db_CD36_T7pR	**TAATACGACTCACTATAGGGG**CATTTCCGGTTGGACATCGATG					
	CD36_cF	ATGCAGAACAAACGGGGG	259	RT-PCR	1.982		
	CD36_qR	ACTCCTCCGCGTTGGTGAC					
draper	c_draper_F	ATGCTGCAGGTCTCAGCAATG	3003	cloning	-	pMINIT_2.0	pMINIT_2.0_draper
	c_draper_R	CTACTCCGGTAGGTTAGGGT					
	T7_draper_F	**TAATACGACTCACTATAGGGG**GTTGAGATCAGCTTCCCTG	566	dsRNA synthesis	-		
	T7_draper_R	**TAATACGACTCACTATAGGGG**GTGAAATATGCCCAGAAGG					
	c_draper_F	ATGCTGCAGGTCTCAGCAATG	207	RT-PCR	1.952		
	q_draper_R	GACTTTGTACCTGGAGCAC					
uk3	c_UK1_f2	CAGCTCATTGACACTACCATG	1370	cloning	-	pMINIT_2.0	pMINIT_2.0_uk1
	c_UK1_r2	GTTTTTGGTTTAGGAGGTCATAC					
	T7_F_UK1	**TAATACGACTCACTATAGGGG**GCGAATCTTGCGACCAACAC	750	dsRNA synthesis	-		
	T7_R_UK1	**TAATACGACTCACTATAGGGG**GGACACCTCGGCGATCACC					
	q_F_UK1	GGCTGGCCTGCGACCATTTC	232	RT-PCR	1.832		
	c_UK1_r2	GTTTTTGGTTTAGGAGGTCATAC					
integrin β	c_IntegrinB_F	ATGAAGATGGAGGTGAGACT	2470	cloning	-	pMINIT_2.0	pMINIT_2.0_integrinB
	c_IntegrinB_R	ATCATTTCCCAGCGTATGTG					
	T7_intB_F	**TAATACGACTCACTATAGGGG**GCGAATCGGCAGTCATCTTC	460	dsRNA synthesis	-		
	T7_intB_R	**TAATACGACTCACTATAGGGG**AGTTCTGCGAGTGTGACAAC					
	q_intB_F9	GCGACGTGTACTCGTTGTC	256	RT-PCR	1.854		
	c_IntegrinB_F	ATGAAGATGGAGGTGAGACT					
fasciclin	c_fasciclin_F	CTCCGCAGCAGACAAAG	1886	cloning	-	pMINIT_2.0	pMINIT_2.0_fasciclin
	c_fasciclin_R2	CAGCATTATCCTTCTGTCAG					
	T7_fasciclin_Ev_F	**TAATACGACTCACTATAGGGG**AGAGTCAACATCTACCCG	578	dsRNA synthesis	-		
	T7_fasciclin_Ev_R	**TAATACGACTCACTATAGGGG**GTGGTGTGGTTAGACGA					
	c_fasciclin_F	CTCCGCAGCAGACAAAG	220	RT-PCR	1.922		
	q_fasciclin_R	CTTCATGGCCTCGTCTAC					
cueball	c_cueball_ Ev_F	CATGAAAGCCGAAAATTC	1818	cloning	-	pMINIT_2.0	pMINIT_2.0_cueball
	c_cueball_Ev_R	CAAGGTCAGTAAAGGTC					
	T7_cueball_Ev_F	**TAATACGACTCACTATAGGGG**GGCGAGAGGTGTGAGATC	508	dsRNA synthesis	-		
	T7_cueball_Ev_R	**TAATACGACTCACTATAGGGG**CCATATTTCCTAAAAGCCCTC					
	qF cueball	GCACCTCTCACTATTCCAG	298	RT-PCR	1.953		
	c_cueball_ Ev_F	CGACCAGAAGACAGACCGA					
Na/Ca exchanger	c_NaCaex_F	GATGTGATGAGCCACGGTC	2582	cloning	-	pMINIT_2.0	pMINIT_2.0_Na/Caexch
	c_NaCaex_R	TCAGAAGTTGAATGGCAGTAC					
	T7_Na/Ca_exchanger_Ev_F	**TAATACGACTCACTATAGGGG**CGGTTCAAGGTTTTCATCG	524	dsRNA synthesis	-		
	T7_Na/Ca_exchanger_Ev_R	**TAATACGACTCACTATAGGGG**GCTGTTGTCTCTTTGTGCTC					
	q_NaCaex_F	GCCGTGTACCACTTTAGC	264	RT-PCR	1.875		
	c_NaCaex_R	TCAGAAGTTGAATGGCAGTAC					
