# Supplementary material for: A knockdown gene approach identifies an insect vector membrane protein with leucin-rich repeats as one of the receptors for the VmpA adhesin of flavescence dorée phytoplasma
Source: Front Cell Infect Microbiol. 2023 Nov 6;13:1289100. doi: 10.3389/fcimb.2023.1289100 (PMC10662966; doi:10.3389/fcimb.2023.1289100)
Supplement: Supplementary file 6 [file DataSheet_6.pdf]

Supplementary Table 2. Euva proteins that interact with VmpA-His$_6$, and have TMHMM-predicted transmembrane segments and ‘NxS/T’ patterns. The proteins selected in this study are in bold and underlined in grey.

Band	TSA *Euscelidius variegatus* (NCBI)	Séquence (BLASTP)	Predicted transmembrane segments (TMHMM)	NxS/T
A	ORF03088356_GFTU01011649.1_00277_02130_f1	PREDICTED: Homalodisca vitripennis ATPase family AAA domain-containing protein 3A homolog	1	1
	ORF03862500_GFTU01015892.1_00313_02331_f1	transmembrane protein 214-B isoform X1 [Homalodisca vitripennis]	1	2
	ORF03578890_GFTU01014139.1_00115_01908_f1	calnexin-like [Homalodisca vitripennis]	1	2
	**ORF03010044_GFTU01011408.1_00185_01996_f2**	**protein cueball isoform X1 [Homalodisca vitripennis]**	**1**	**6**
	ORF03468514_GFTU01013357.1_00081_01916_f3	dolichyl-diphosphooligosaccharide--protein glycosyltransferase subunit 1 [Homalodisca vitripennis]	1	2
	ORF01460866_GFTU01005403.1_00284_02236_f2	uncharacterized protein LOC106664528 isoform X3 [Cimex lectularius]	1	3
	ORF03397146_GFTU01012809.1_00455_02434_f2	T-cell immunomodulatory protein isoform X4 [Homalodisca vitripennis]	2	5
	ORF02280746_GFTU01008614.1_00587_02362_f2	hypothethical protein Homalodisca vitripennis LOC124363104 isoform X2 and J6590_050414	1	4
	**ORF01214079_GFTU01004399.1_00136_01779_f1**	**lysosome membrane protein 2 [Homalodisca vitripennis]**	**2**	**6**
	ORF00500949_GFTU01001590.1_00286_02226_f1	polypeptide N-acetylgalactosaminyltransferase 1-like isoform X1 [Homalodisca vitripennis]	1	2
	ORF01424621_GFTU01005272.1_00244_01983_f1	plexin domain-containing protein 2-like isoform X1 [Homalodisca vitripennis]	1	8
	ORF01046095_GFTU01003760.1_00286_02106_f1	polypeptide N-acetylgalactosaminyltransferase 5 isoform X1 [Homalodisca vitripennis]	1	2
	ORF03532966_GFTU01013866.1_00112_01845_f1	golgin (Homalodisca vitripennis)	1	3
	ORF01840417_GFTU01006919.1_00002_01687_f2	hypothetical protein H. viripennis	10	2
	ORF00137267_GFTU01000459.1_00003_01973_f3	uncharacterized protein LOC124354663 isoform X1 [Homalodisca vitripennis]	1	3
	ORF00766636_GFTU01002643.1_00132_01904_f3	D-glucuronyl C5-epimerase [Homalodisca vitripennis]	1	3
	ORF01956414_GFTU01007342.1_00121_02085_f1	dolichyl-diphosphooligosaccharide--protein glycosyltransferase subunit 2 [Homalodisca vitripennis]	3	2
	ORF01252837_GFTU01004577.1_00244_02139_f1	N-acetylgalactosaminyltransferase 6-like isoform X1 [Homalodisca vitripennis]	1	2
	ORF03537334_GFTU01013894.1_00064_02025_f1	hypothetical protein J6590_074417 [Homalodisca vitripennis]	2	4
	ORF03590659_GFTU01014209.1_00312_02150_f3	neutral and basic amino acid transport protein rBAT-like [Homalodisca vitripennis]	1	9
	ORF03587765_GFTU01014197.1_00076_02181_f1	nicastrin isoform X1 [Homalodisca vitripennis]	1	11
B	ORF03693862_GFTU01014848.1_00369_01463_f3	heparan sulfate 2-O-sulfotransferase 1-like [Homalodisca vitripennis]	1	2
	ORF03657744_GFTU01014605.1_00086_01171_f2	prohibitin-2-like [Homalodisca vitripennis]	1	1
	ORF00313454_GFTU01000993.1_00057_01349_f3	GDP-fucose protein O-fucosyltransferase 1-like [Homalodisca vitripennis]	1	1
	ORF00775440_GFTU01002668.1_00113_01303_f2	glutaminyl-peptide cyclotransferase-like [Homalodisca vitripennis]	1	1
	ORF00582827_GFTU01001844.1_00001_01110_f1	basigin isoform X2 [Homalodisca vitripennis]	1	6
	ORF00738628_GFTU01002531.1_00152_01321_f2	dnaJ homolog subfamily B member 14 [Homalodisca vitripennis]	1	2
	ORF03486590_GFTU01013538.1_00143_01228_f2	innexin inx2 [Homalodisca vitripennis]	4	1
	ORF02385866_GFTU01009016.1_00139_01251_f1	acyl-CoA Delta-9 desaturase-like [Homalodisca vitripennis]	4	3
	ORF04030053_GFTU01016892.1_00210_01373_f3	cysteine-rich with EGF-like domain protein 2 [Homalodisca vitripennis]	2	2
	ORF01207302_GFTU01004379.1_00053_01219_f2	SLIT and NTRK-like protein 4 [Homalodisca vitripennis]	1	2
	ORF03273504_GFTU01012324.1_00179_01489_f2	protein catecholamines up-like [Homalodisca vitripennis]	6	1
	ORF04005737_GFTU01016727.1_00069_01361_f3	V-type proton ATPase subunit S1 [Homalodisca vitripennis]	1	4
	ORF02908881_GFTU01010984.1_00080_01198_f2	solute carrier family 35 member F6 [Homalodisca vitripennis]	10	1
	ORF03617956_GFTU01014363.1_00184_01326_f1	ceramide synthase 6 [Homalodisca vitripennis]	7	2
	ORF01236041_GFTU01004522.1_00348_01424_f3	galactosylgalactosylxylosylprotein 3-beta-glucuronosyltransferase I-like isoform X1 [Homalodisca vitripennis]	1	2
	ORF03436733_GFTU01013123.1_00289_01524_f1	peptidyl-prolyl cis-trans isomerase FKBP8 [Homalodisca vitripennis]	1	4
	ORF02798948_GFTU01010594.1_00261_01460_f3	acid phosphatase like	1	2
C	ORF01780037_GFTU01006645.1_00109_01017_f1	integral membrane protein 2B-like [Homalodisca vitripennis]	1	2
	ORF03212397_GFTU01012093.1_00208_01215_f1	vesicular integral-membrane protein VIP36-like [Homalodisca vitripennis]	1	2
	ORF01363517_GFTU01005045.1_00122_01018_f2	syntaxin isoform X3 [Frankliniella occidentalis]	1	3
	ORF02444589_GFTU01009226.1_00223_01083_f1	voltage-dependent anion channel [Recilia dorsalis]/putative mitochondrial porin [Homalodisca vitripennis]	18	4
	ORF03099463_GFTU01011702.1_00153_01151_f3	apolipoprotein D-like [Homalodisca vitripennis]	1	1
	ORF03885779_GFTU01016029.1_00130_00987_f1	Syntaxin [Homalodisca vitripennis]	1	1
	ORF00932050_GFTU01003335.1_00105_01109_f3	renin receptor [Homalodisca vitripennis] /ATPase H(+)-transporting accessory protein 2 [Homalodisca vitripennis]	1	2
	ORF01417067_GFTU01005233.1_00001_00918_f1	prohibitin [Acyrthosiphon pisum]	1	1
	ORF02696019_GFTU01010220.1_00149_01096_f2	protein AGAP004871-like isoform X3 [Schistocerca americana]	2	6
	ORF01807241_GFTU01006798.1_00091_00969_f1	DDRGK domain-containing protein 1, variant 2 [Homalodisca vitripennis]	1	1
	ORF00675649_GFTU01002260.1_00154_01077_f1	SWI/SNF and RSC complex subunit Ssr1 [Homalodisca vitripennis]	1	3
	ORF03414034_GFTU01012926.1_00456_01385_f3	N-acetylglucosaminyl-phosphatidylinositol de-N-acetylase-like isoform X1 [Homalodisca vitripennis]	1	3
	ORF03229291_GFTU01012148.1_00149_01072_f2	ribonuclease Oy [Homalodisca vitripennis]	1	5
	ORF01846268_GFTU01006941.1_00102_01085_f3	decorin [Homalodisca vitripennis]	1	4
D	ORF01649733_GFTU01006110.1_00059_00856_f2	type 1 phosphatidylinositol 4,5-bisphosphate 4-phosphatase isoform X1 [Homalodisca vitripennis]	2	1
	ORF01650080_GFTU01006112.1_00156_00785_f3	transmembrane emp24 domain-containing protein-like [Homalodisca vitripennis]	1	1
	ORF00042812_GFTU01000143.1_00091_00750_f1	TMEM9/TMEM9B (identification *via* interpro)	1	3
	ORF04071547_GFTU01017136.1_00109_00867_f1	vesicle-associated membrane protein/synaptobrevin-binding protein [Homalodisca vitripennis]	1	1
	ORF02665838_GFTU01010090.1_00258_00932_f3	dnaJ homolog subfamily C member 5 isoform X3 [Homalodisca vitripennis]	2	1
	ORF02665501_GFTU01010089.1_00258_00992_f3	dnaJ homolog subfamily C member 5 isoform X2 [Homalodisca vitripennis]	2	1
	ORF00874963_GFTU01003092.1_00327_01094_f3	signal recognition particle receptor subunit beta-like [Homalodisca vitripennis]	1	1
	ORF01219637_GFTU01004432.1_00220_00822_f1	translocon-associated protein subunit beta [Homalodisca vitripennis]	1	2
	ORF01521166_GFTU01005634.1_00017_00550_f2	STI1-like protein [Homalodisca vitripennis]	2	2
	ORF00820901_GFTU01002861.1_00114_00920_f3	aquaporin AQPAe.a isoform X2 [Homalodisca vitripennis]	6	1
	ORF00821078_GFTU01002862.1_00114_00908_f3	aquaporin AQPAe.a isoform X4 [Homalodisca vitripennis]	6	1
	ORF03776752_GFTU01015372.1_00129_00854_f3	syntaxin-8 [Homalodisca vitripennis]	1	1
	ORF03420291_GFTU01012977.1_00417_01208_f3	CD63 antigen [Nephotettix cincticeps]	4	4
	ORF03441112_GFTU01013144.1_00157_00810_f1	vesicle-trafficking protein SEC22b [Homalodisca vitripennis]	1	2
	ORF01794428_GFTU01006722.1_00175_00837_f1	transmembrane emp24 domain-containing protein 7 [Homalodisca vitripennis]	1	1
	ORF02060243_GFTU01007701.1_00290_01039_f2	vesicle transport protein USE1 isoform X1 [Homalodisca vitripennis]	1	2
E	ORF03557296_GFTU01013999.1_00232_00624_f1	uncharacterised proteins	4	2
F	ORF03591272_GFTU01014213.1_00151_03195_f1	calcium-transporting ATPase sarcoplasmic/endoplasmic reticulum type isoform X3 [Cimex lectularius]	10	5
	ORF02774007_GFTU01010510.1_00279_03419_f3	sodium/potassium-transporting ATPase subunit alpha isoform X1 [Homalodisca vitripennis]	10	5
	ORF01543048_GFTU01005721.1_00407_03304_f2	lysosomal alpha-glucosidase-like [Homalodisca vitripennis]	1	13
	ORF04029061_GFTU01016887.1_00003_02834_f3	aminopeptidase-like protein [Homalodisca vitripennis]	1	5
	ORF00672315_GFTU01002249.1_00136_03036_f1	fasciclin-2-like [Homalodisca vitripennis]	2	7
	ORF00224802_GFTU01000703.1_01002_04178_r3	uncharacterized protein LOC124364686 [Homalodisca vitripennis]	1	13
	ORF02806491_GFTU01010616.1_00518_03514_f2	lysosomal alpha-glucosidase-like [Homalodisca vitripennis]	1	14
	ORF00577827_GFTU01001820.1_00276_03056_f3	uncharacterized protein LOC111864751 isoform X1 [Cryptotermes secundus]	1	4
	ORF01146851_GFTU01004135.1_00123_02915_f3	ER membrane protein complex subunit 1 [Homalodisca vitripennis]	1	4
	**ORF03898939_GFTU01016123.1_00223_03282_f1**	**protein draper isoform X1 [Zootermopsis nevadensis]**	**1**	**11**
	**ORF02093016_GFTU01007852.1_00001_02736_f1**	**transforming growth factor-beta-induced protein ig-h3-like [Homalodisca vitripennis]**	**1**	**5**
	ORF00406425_GFTU01001302.1_00108_03002_f3	calcium-transporting ATPase type 2C member 1-like [Homalodisca vitripennis]	10	4
	ORF01573661_GFTU01005820.1_00001_03258_f1	NAD(P) transhydrogenase, mitochondrial-like isoform X1 [Homalodisca vitripennis]	14	7
G+H	ORF01500200_GFTU01005554.1_00297_02693_f3	PREDICTED: Homalodisca vitripennis neurotactin-like (LOC124361907), mRNA	1	8
	**ORF03757228_GFTU01015247.1_00222_02681_f3**	**Protein containing LRR domains (identification via interpro)**	**1**	**14**
	**ORF03985247_GFTU01016611.1_00180_02645_f3**	**integrin beta**	**1**	**7**
	ORF01543048_GFTU01005721.1_00407_03304_f2	lysosomal alpha-glucosidase-like [Homalodisca vitripennis]	1	15
	**ORF03874906_GFTU01015965.1_00117_02747_f3**	**sodium/calcium exchanger 3-like isoform X1 [Homalodisca vitripennis]**	**12**	**6**
	ORF03008926_GFTU01011402.1_00250_02643_f1	Gelsolin, cytoplasmic [Cryptotermes secundus]	1	2
	ORF02638676_GFTU01009977.1_00162_02546_f3	AFG3-like protein 2 [Homalodisca vitripennis]	2	3
	ORF01921174_GFTU01007221.1_00361_02811_f1	mannosyl-oligosaccharide glucosidase-like [Homalodisca vitripennis]	1	4
	**ORF02093016_GFTU01007852.1_00001_02736_f1**	**transforming growth factor-beta-induced protein ig-h3-like [Homalodisca vitripennis]**	**1**	**5**
	ORF01868234_GFTU01007015.1_00149_02551_f2	extended synaptotagmin-2 isoform X3 [Homalodisca vitripennis]	2	5
	ORF01146851_GFTU01004135.1_00123_02915_f3	ER membrane protein complex subunit 1 [Homalodisca vitripennis]	1	4
	**ORF03639633_GFTU01014488.1_00117_02468_f3**	**leucine-rich repeat-containing protein 15-like [Homalodisca vitripennis]**	**1**	**7**
	ORF02805699_GFTU01010613.1_00159_02690_f3	mitochondrial proton/calcium exchanger protein [Homalodisca vitripennis]	2	3
	ORF00406425_GFTU01001302.1_00108_03002_f3	calcium-transporting ATPase type 2C member 1-like [Homalodisca vitripennis]	10	4
I	ORF03655252_GFTU01014587.1_00207_01721_f3	tumor necrosis factor receptor superfamily member wengen [Anoplophora glabripennis]	1	2
	ORF01196773_GFTU01004342.1_00189_01889_f3	stromal interaction molecule homolog isoform X2 [Homalodisca vitripennis]	2	2
	ORF04114081_GFTU01017546.1_00249_02021_f3	N-acetylgalactosaminyltransferase 7 [Homalodisca vitripennis]	1	2
	ORF03358881_GFTU01012628.1_00448_01920_f1	protein croquemort-like isoform X1 [Homalodisca vitripennis]	2	8
	ORF03008216_GFTU01011397.1_00217_01842_f1	probable cytochrome P450 6d5 [Homalodisca vitripennis]	1	1
	ORF03185639_GFTU01012013.1_00219_01796_f3	putative fatty acyl-CoA reductase CG8306 isoform X1 [Homalodisca vitripennis]	2	2
	ORF01900380_GFTU01007128.1_00486_02108_f3	KAG8267176.1 hypothetical protein J6590_057015 [Homalodisca vitripennis]	2	3
	ORF03737405_GFTU01015141.1_00137_01597_f2	cytochrome P450 2C31-like isoform X1 [Homalodisca vitripennis]	1	3
	ORF03903967_GFTU01016147.1_00084_01781_f3	proton-coupled amino acid transporter-like protein pathetic isoform X3 [Halyomorpha halys]	11	7
	ORF00673844_GFTU01002254.1_00165_01679_f3	X-linked interleukin-1 receptor accessory protein-like 2 [Homalodisca vitripennis]	1	8
	ORF01527703_GFTU01005654.1_00096_01697_f3	heme binding [Homalodisca vitripennis]	1	6
	ORF01668027_GFTU01006187.1_00257_01861_f2	UDP-glycosyltransferase UGT4-like [Homalodisca vitripennis]	1	2
	ORF03712827_GFTU01014988.1_00517_02010_f1	Golgi integral membrane protein 4-like isoform X1 [Homalodisca vitripennis]	1	1
	ORF03444976_GFTU01013184.1_00146_01954_f2	zinc transporter foi-like isoform X2 [Homalodisca vitripennis]	6	7
	ORF01840417_GFTU01006919.1_00002_01687_f2	delta(14)-sterol reductase TM7SF2 [Homalodisca vitripennis]	10	2
	**ORF03010044_GFTU01011408.1_00185_01996_f2**	**protein cueball isoform X1 [Homalodisca vitripennis]**	**1**	**6**
	ORF02440449_GFTU01009199.1_00119_01927_f2	GPI transamidase component PIG-T-like [Homalodisca vitripennis]	2	8
	ORF01967244_GFTU01007378.1_00019_01656_f1	amyloid precursor protein metabolic process [Homalodisca vitripennis]	3	3
	ORF01126207_GFTU01004066.1_00444_02081_f3	glutathione hydrolase 7-like isoform X1 [Homalodisca vitripennis]	1	5
	ORF04139556_GFTU01017799.1_00052_01656_f1	chondroadherin-like [Homalodisca vitripennis]	1	4
	ORF01424621_GFTU01005272.1_00244_01983_f1	plexin domain-containing protein 2-like isoform X1 [Homalodisca vitripennis]	1	8
	ORF03532966_GFTU01013866.1_00112_01845_f1	golgin-84-like [Homalodisca vitripennis]	1	3
	ORF03433846_GFTU01013097.1_00226_01893_f1	nicalin-1-like [Homalodisca vitripennis]	1	3
	ORF00064229_GFTU01000227.1_00351_01874_f3	activin receptor type-2A [Nilaparvata lugens]	1	4
	ORF01224055_GFTU01004452.1_00050_01720_f2	G-protein coupled receptor Mth2-like isoform X2 [Homalodisca vitripennis]	8	6
	ORF04034680_GFTU01016922.1_00055_01536_f1	probable cytochrome P450 6a13 [Homalodisca vitripennis]	1	1
	ORF03339069_GFTU01012554.1_00002_01780_f2	activin receptor type-1 isoform X1 [Zootermopsis nevadensis]	1	3
	ORF02142225_GFTU01008058.1_00314_01939_f2	protein wntless [Homalodisca vitripennis]	8	5
	ORF01126404_GFTU01004067.1_00066_01775_f3	Homalodisca vitripennis probable cytochrome P450 6d5 (LOC124365725), mRNA	1	1
	ORF03729559_GFTU01015089.1_00480_02093_f3	insulin-like growth factor-binding protein complex acid labile subunit [Homalodisca vitripennis]	1	3
	**ORF01214079_GFTU01004399.1_00136_01779_f1**	**lysosome membrane protein 2 [Homalodisca vitripennis]**	**2**	**6**
	ORF03944081_GFTU01016384.1_00181_01875_f1	PREDICTED: Cryptotermes secundus protein ST7 homolog (LOC111871312), transcript variant X1, mRNA	3	2
	ORF00487573_GFTU01001542.1_00181_01770_f1	UDP-glycosyltransferase UGT5-like isoform X1 [Homalodisca vitripennis]	1	2
	ORF03578890_GFTU01014139.1_00115_01908_f1	PREDICTED: Homalodisca vitripennis calnexin-like (LOC124358330), mRNA	1	2
	ORF02735221_GFTU01010376.1_00120_01757_f3	probable cytochrome P450 6a14 [Homalodisca vitripennis]	1	4
	ORF00707446_GFTU01002392.1_00094_01698_f1	UDP-glycosyltransferase UGT5 isoform X3 [Nilaparvata lugens]	1	4
	ORF00954693_GFTU01003435.1_00111_01808_f3	protein spinster isoform X2 [Homalodisca vitripennis]	12	4
	ORF00831169_GFTU01002905.1_00011_01504_f2	UDP-GalNAc:beta-1,3-N-acetylgalactosaminyltransferase 2-like isoform X1 [Homalodisca vitripennis]	1	5
	ORF00852307_GFTU01002995.1_00138_01805_f3	putative fatty acyl-CoA reductase CG5065 [Homalodisca vitripennis]	1	2
	ORF02295880_GFTU01008690.1_00240_02024_f3	melanocyte protein PMEL isoform X1 [Homalodisca vitripennis]	1	13
	ORF01632836_GFTU01006031.1_00129_01964_f3	PREDICTED: Homalodisca vitripennis facilitated trehalose transporter Tret1-2 homolog (LOC124354290), mRNA	12	1
	ORF02260132_GFTU01008545.1_00206_01705_f2	uncharacterized protein LOC124364991 isoform X1 [Homalodisca vitripennis]	1	7
	ORF00875556_GFTU01003096.1_00337_01950_f1	cleft lip and palate transmembrane protein 1-like protein [Nilaparvata lugens]	8	2
	ORF02363633_GFTU01008940.1_00356_01870_f2	major facilitator superfamily domain-containing protein 1-like [Homalodisca vitripennis]	12	6
	ORF02945676_GFTU01011139.1_00265_01836_f1	equilibrative nucleoside transporter 1 [Homalodisca vitripennis]	11	3
	ORF00772888_GFTU01002658.1_00178_01728_f1	PREDICTED: Bemisia tabaci protein halfway (LOC109035584), mRNA	1	8
	ORF03488356_GFTU01013548.1_00103_01815_f1	protein GPR107 [Homalodisca vitripennis]	7	3
	ORF00935122_GFTU01003349.1_00078_01886_f3	protein Malvolio-like isoform X5 [Homalodisca vitripennis]	12	3
	ORF03297927_GFTU01012417.1_00170_01831_f2	PREDICTED: Homalodisca vitripennis probable cytochrome P450 6a14 (LOC124355178), mRNA	1	1
	ORF02444245_GFTU01009225.1_00180_01838_f3	protein brambleberry-like [Homalodisca vitripennis]	3	3
	ORF01995855_GFTU01007457.1_00195_01676_f3	diacylglycerol O-acyltransferase 1 isoform X1 [Nilaparvata lugens]	9	1
	ORF03999079_GFTU01016699.1_00001_01743_f1	monocarboxylate transporter 13 isoform X1 [Homalodisca vitripennis]	12	8
	ORF00296300_GFTU01000932.1_00179_01981_f2	organic cation transporter protein isoform X1 [Homalodisca vitripennis]	12	5
	ORF03881511_GFTU01016002.1_00246_01973_f3	tyrosine-protein kinase Dnt [Homalodisca vitripennis]	1	6
J	ORF04005737_GFTU01016727.1_00069_01361_f3	V-type proton ATPase subunit S1 [Homalodisca vitripennis]	1	4
	ORF03540683_GFTU01013912.1_00212_01462_f2	endoplasmic reticulum junction formation protein lunapark-B isoform X5 [Homalodisca vitripennis]	2	1
	ORF02853024_GFTU01010796.1_00067_01248_f1	translocation protein SEC62 [Homalodisca vitripennis]	2	1
	ORF00057085_GFTU01000202.1_00329_01771_f2	calcium-binding mitochondrial carrier protein SCaMC-2 isoform X1 [Homalodisca vitripennis]	2	3
	ORF03087292_GFTU01011645.1_00253_01608_f1	uncharacterized protein LOC124354859 [Homalodisca vitripennis]	1	2
	ORF03436733_GFTU01013123.1_00289_01524_f1	Homalodisca vitripennis peptidyl-prolyl cis-trans isomerase FKBP8	1	4
	ORF02125708_GFTU01007996.1_00254_01681_f2	serine palmitoyltransferase 1 [Homalodisca vitripennis]	1	2
	ORF03470760_GFTU01013384.1_00142_01362_f1	acyl-CoA-binding domain-containing protein 4 [Homalodisca vitripennis]	1	3
	ORF00490218_GFTU01001550.1_00162_01538_f3	fasciclin-3 [Halyomorpha halys]	1	4
	ORF03518135_GFTU01013773.1_00156_01433_f3	protein disulfide-isomerase TMX3 [Homalodisca vitripennis]	1	3
	ORF02184618_GFTU01008239.1_00219_01595_f3	alpha-1,3-mannosyl-glycoprotein 2-beta-N-acetylglucosaminyltransferase-like [Homalodisca vitripennis]	1	2
	ORF00202071_GFTU01000624.1_00003_01334_f3	heparan-sulfate 6-O-sulfotransferase 1 [Homalodisca vitripennis]	1	2
	ORF03486590_GFTU01013538.1_00143_01228_f2	innexin inx2 [Homalodisca vitripennis]	4	1
	ORF00582827_GFTU01001844.1_00001_01110_f1	basigin isoform X2 [Homalodisca vitripennis]	1	6
	ORF01513979_GFTU01005613.1_00316_01674_f1	prostatic acid phosphatase-like isoform X3 [Homalodisca vitripennis]	1	5
	ORF03657744_GFTU01014605.1_00086_01171_f2	prohibitin-2-like [Homalodisca vitripennis]	1	1
	ORF01076354_GFTU01003873.1_00500_01660_f2	no identification even with interpro uncharacterized protein LOC124366327 [Homalodisca vitripennis]	1	4
	ORF03384640_GFTU01012731.1_00475_01770_f1	beta-1,4-glucuronyltransferase 1-like [Homalodisca vitripennis]	1	1
	ORF02660245_GFTU01010066.1_00198_01325_f3	glycoprotein-N-acetylgalactosamine 3-beta-galactosyltransferase 1-like [Homalodisca vitripennis]	1	2
	ORF02385866_GFTU01009016.1_00139_01251_f1	acyl-CoA Delta-9 desaturase-like [Homalodisca vitripennis]	4	3
	ORF03511067_GFTU01013724.1_00051_01310_f3	uncharacterized protein LOC124365941 [Homalodisca vitripennis]	1	5
	ORF03871669_GFTU01015944.1_00414_01907_f3	facilitated trehalose transporter Tret1-like isoform X1 [Homalodisca vitripennis]	12	1
	ORF00914527_GFTU01003269.1_00241_01365_f1	lactosylceramide 4-alpha-galactosyltransferase isoform X2 [Nilaparvata lugens]	1	3
	ORF00748024_GFTU01002569.1_00005_01330_f2	minor histocompatibility antigen H13 isoform X2 [Homalodisca vitripennis]	9	3
	ORF03178020_GFTU01011979.1_00333_01703_f3	transducin beta-like protein 2 [Homalodisca vitripennis]	1	1
	ORF01842823_GFTU01006929.1_00321_01577_f3	soluble calcium-activated nucleotidase 1 [Homalodisca vitripennis]	1	2
	ORF00936187_GFTU01003353.1_00246_01613_f3	sorting and assembly machinery component 50 homolog B [Homalodisca vitripennis]	16	3
	ORF03666283_GFTU01014673.1_00036_01256_f3	cuticlin-4 [Homalodisca vitripennis]	1	2
	ORF00120350_GFTU01000411.1_00625_02058_f1	zinc transporter 2 [Homalodisca vitripennis]	6	3
	ORF03680891_GFTU01014752.1_00164_01375_f2	PREDICTED: Homalodisca vitripennis translocating chain-associated membrane protein 1	8	3
	ORF03283506_GFTU01012354.1_00299_01654_f2	cystinosin homolog isoform X2 [Nilaparvata lugens]	7	3
	ORF03510638_GFTU01013721.1_00044_01216_f2	growth hormone-inducible transmembrane protein-like [Homalodisca vitripennis]	9	1
	ORF02861965_GFTU01010826.1_00151_01230_f1	cell cycle control protein 50A isoform X4 [Homalodisca vitripennis]	2	3
	ORF02908881_GFTU01010984.1_00080_01198_f2	solute carrier family 35 member F6 [Homalodisca vitripennis]	10	1
	ORF01227052_GFTU01004467.1_00235_01416_f1	PREDICTED: Homalodisca vitripennis lysophosphatidylserine lipase ABHD12-like (LOC124364729), transcript variant X2, mRNA	1	4
	ORF01543686_GFTU01005723.1_00001_01446_f1	prolactin regulatory element-binding protein [Homalodisca vitripennis]	1	3
	ORF00624853_GFTU01002044.1_00310_01548_f1	glycosylated lysosomal membrane protein-like [Homalodisca vitripennis]	1	11
	ORF01384059_GFTU01005117.1_00356_01645_f2	zinc/cadmium resistance protein [Homalodisca vitripennis]	6	1
	ORF00775000_GFTU01002666.1_00231_01415_f3	PREDICTED: innexin inx3 [Bemisia tabaci]	4	1
	ORF01046563_GFTU01003761.1_00147_01544_f3	protein odr-4 homolog [Homalodisca vitripennis]	1	3
	ORF03273504_GFTU01012324.1_00179_01489_f2	protein catecholamines up-like [Homalodisca vitripennis]	6	1
	ORF01678053_GFTU01006215.1_00001_01314_f1	ER lumen protein-retaining receptor [Homalodisca vitripennis]	6	3
	ORF01929554_GFTU01007246.1_00125_01513_f2	UNC93-like protein MFSD11 isoform X1 [Homalodisca vitripennis]	12	3
	ORF03110745_GFTU01011746.1_00300_01667_f3	uncharacterized protein LOC124357838 [Homalodisca vitripennis]	1	6
	ORF03791453_GFTU01015463.1_00172_01353_f1	endoplasmic reticulum-Golgi intermediate compartment protein 3-like isoform X1 [Homalodisca vitripennis]	2	4
	ORF02798948_GFTU01010594.1_00261_01460_f3	PREDICTED: Homalodisca vitripennis prostatic acid phosphatase-like (LOC124371213), mRNA	1	2
	ORF02848509_GFTU01010777.1_00460_01722_f1	PREDICTED: Homalodisca vitripennis endoplasmic reticulum-Golgi intermediate compartment protein 2 (LOC124364981), mRNA	2	1
	ORF03748447_GFTU01015190.1_00026_01183_f2	fat storage-inducing transmembrane protein 2 [Homalodisca vitripennis]	6	1
	ORF03509806_GFTU01013713.1_00318_01721_f3	UNC93-like protein MFSD11 [Homalodisca vitripennis]	12	1
	ORF01844038_GFTU01006936.1_00254_01579_f2	PREDICTED: Homalodisca vitripennis transmembrane protein 268-like (LOC124364903), mRNA	2	3
	ORF03617956_GFTU01014363.1_00184_01326_f1	ceramide synthase 6 [Homalodisca vitripennis]	7	2
	**ORF00560024_GFTU01001746.1_00401_01750_f2**	**uncharacterized protein LOC124356975 [Homalodisca vitripennis]**	**1**	**2**
	ORF03616652_GFTU01014357.1_00277_01377_f1	protein YIF1B [Nilaparvata lugens]	5	2
	ORF00941208_GFTU01003375.1_00255_01664_f3	glycosaminoglycan xylosylkinase [Homalodisca vitripennis]	1	4
	ORF00715920_GFTU01002430.1_00333_01784_f3	PREDICTED: Homalodisca vitripennis acidic mammalian chitinase-like (LOC124362433), transcript variant X1, mRNA	1	6
