# Supplementary material for: A knockdown gene approach identifies an insect vector membrane protein with leucin-rich repeats as one of the receptors for the VmpA adhesin of flavescence dorée phytoplasma
Source: Front Cell Infect Microbiol. 2023 Nov 6;13:1289100. doi: 10.3389/fcimb.2023.1289100 (PMC10662966; doi:10.3389/fcimb.2023.1289100)
Supplement: Supplementary file 7 [file DataSheet_7.pdf]

Supplementary Table 3. Homologs of uk1_LRR in NCBI databases using Blast and domains found with InterPro.

	Blast output						interpro prediction	
**description**	**Blast**	**organism**	**query cover (%)**	**E value**	**identity %**	**accession number**	**number of LRR domains**	**type of LRR domains**
carboxypeptidase N subnit 2-like	blastn	*Macrosteles quadrilineautus*	84	0.0	81.23	XM_054410529.1<br>XM_054410528.1<br>XM_054410527.1	no prediction<br>no prediction<br>no prediction	no prediction<br>no prediction<br>no prediction
carboxypeptidase N subnit 2-like	blastp	*Macrosteles quadrilineautus*	99	0.0	91.63	XP_054266502.1	no prediction	no prediction
protein artichoke-like	blastp	*Homalodisca vitripennis*	96	0.0	72.33	XP_046674361.1	4	RI
hypothetical protein J6590_064756	blastp	*Homalodisca vitripennis*	88	0.0	73.03	KAG8256621.1	3	RI
Toll pathway protein	blastp	*Laodelphax striatellus*	86	0.0	62.70	AMQ10343.1	5	RI
hypotethical protein LSTR_LSTR014165	blastp	*Laodelphax striatellus*	86	0.0	62.85	RZF39866.1	5	RI
chaoptin	blastp	*Nilaparvata lugens*	86	0.0	62.76	XP_022200047.1	5	RI
PREDICTED: insulin-like growth factor binding protein complex acid labile subunit	blastp	*Bemisia tabaci*	93	0.0	57.44	XP_018904807.1	5	RI
unnamed protein product	blastp	*Bemisia tabaci*	93	0.0	57.30	CAH0754027.1	5	RI
insulin-like growth factor binding protein complex acid labile subunit	blastp	*Halyomorpha halys*	95	0.0	56.13	XP_014293272.1	5	RI
unnamed protein product	blastp	*Nezara viridula*	95	0.0	55.98	CAH1406343.1	5	RI
toll-like receptor Tollo	blastp	*Daktulosphaira vitifoliae*	94	0.0	56.64	XP_050535112.1	5	RI
carboxypeptidase N subunit 2-like	blastp	*Sipha flava*	94	0.0	56.56	XP_025425654.1	5	RI
uncharacterised protein LOC126845587	blastp	*Adelges cooleyi*	86	0.0	59.41	XP_050440269.1	5	RI
hypothetical protein AGLY_013745	blastp	*Aphis glycines*	93	0.0	56.27	KAE9526114.1	5	RI
carboxypeptidase N subunit 2-like	blastp	*Melanaphis sacchari*	94	0.0	56.02	XP_025196330.1	5	RI
